# Activated Carbon Production from Coffee Waste Employing Soil, Mining Waste, and Hematite as Activating Agents

**DOI:** 10.1021/acsomega.6c00989

**Published:** 2026-10-06

**Authors:** Pricila M. B. Chagas, Aline A. Caetano, Evanise S. Penido, Mozarte S. Santana, Maria Das Gracas Cardoso, Fabiano Magalhães

**Affiliations:** Inorganic Chemistry Laboratory, Department of Chemistry, Federal University of Lavras, Lavras, State of Minas Gerais 37203-202, Brazil

## Abstract

Coffee bean cake (CC) is the waste generated after the extraction of syrup from coffee beans. In this study, CC was used as a precursor to prepare activated carbons (AC) using novel Fe-rich activating agents. Initially, the biomass (CC) was pyrolyzed at 450 °C for 90 min to obtain biochar (pyrolyzed coffee cake—PCC). Then, PCC was subjected to activation using three different iron-rich materials: commercial hematite (AC_Fe_2_O_3_), mining waste (AC_waste), and a soil sample (AC_soil). The materials were characterized and used to adsorb paracetamol (acetaminophen). The activated carbons AC_Fe_2_O_3_, AC_soil, and AC_waste showed specific surface areas of 614, 578, and 458 m^2^ g^–1^, respectively, which were nearly 22 times higher when compared with their precursor CC (21 m^2^ g^–1^). In the adsorption experiments of paracetamol, equilibrium was reached in approximately 180 min of reaction with high removal by the prepared activated carbons (AC_Fe_2_O_3_ = 82.5%, AC_soil = 67.1%, and AC_waste = 51.4%) when compared with CC and PCC (4.2% and 3.7%, respectively). The kinetic and adsorption isotherm models suggest that the adsorption of paracetamol by AC_Fe_2_O_3_ may be described by a process predominantly associated with chemisorption, while AC_soil and AC_waste show a better fit to the Freundlich model, which may indicate the occurrence of a multilayer removal mechanism.

## Graphical abstract

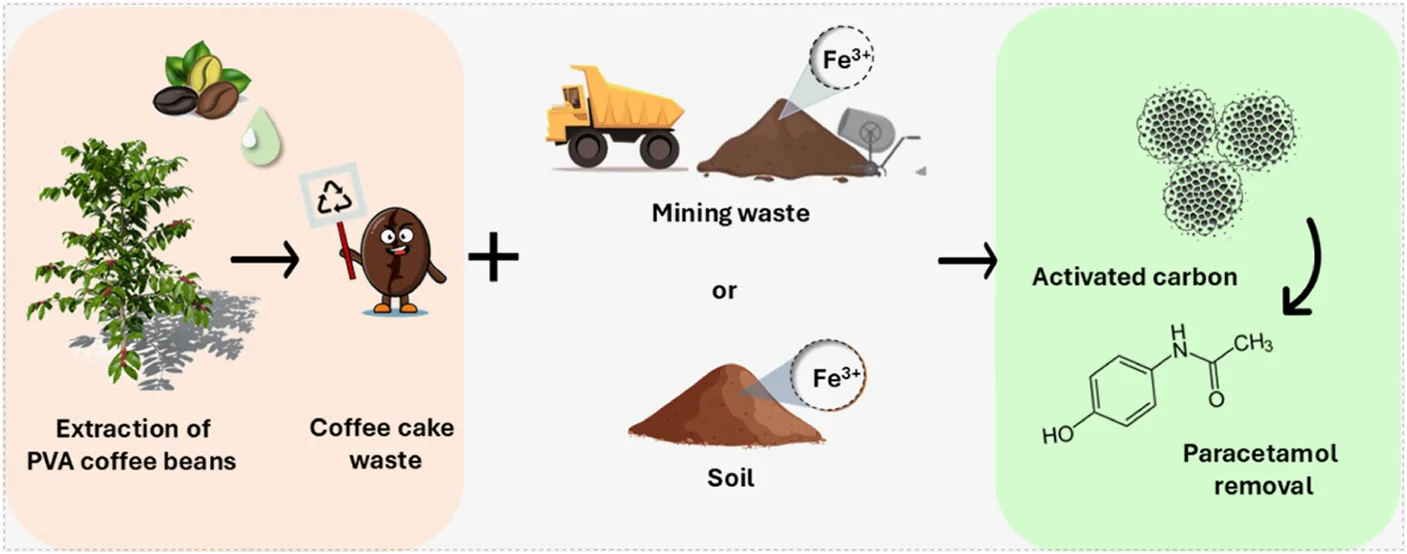

## 1 Introduction

Coffee is the third most consumed beverage in the world. In 2023, global coffee consumption surpassed the 10 billion kilograms mark.^1^ South America plays a central role in this scenario, consolidating itself as the main coffee producing region with a 4.8% increase in production in the 2022/23 coffee year compared to the previous year.^1^ Brazil, in particular, remains the world’s largest coffee exporter with 39.2 million 60 kg bags of coffee shipped to international markets in 2023.^2^

After coffee harvesting and processing stages, substantial volumes of waste are generated. The amount of waste generated in each harvest is proportional to the grain production. Therefore, considering the annual growth in coffee production, a significant increase in the generation of this waste is projected for the coming decades. Agricultural by-products such as those from coffee represent a valuable source for the development of various technological materials, both from an economic and environmental point of view. This is due to its low cost, diverse origin and wide availability.^3^

One of the main residues generated in the coffee sector consists of low-quality and defective beans.^4^ Among the defects that most impact cup quality are black, green (immature or unripe), and sour beans.^5^ The average proportion of defective beans can reach up to 20% of the total production, depending on harvesting conditions.^6^ One of the alternatives considered for the reuse of these beans is the extraction of oils for the production of biodiesel and syrups used in the production of food matrices, pharmaceuticals and cosmetics.^7,8^ However, these processes generate a solid by-product rich in lignocellulose, known as coffee cake (CC), which also has significant potential for reuse, allowing the cycle to be closed with the full reuse of the residue.

A promising alternative for the reuse of CC is the production of activated carbon, a highly porous material obtained from a carbon-rich precursor using a chemical or physical activation process.^9–11^ The chemical activation method is particularly effective for producing materials with a more developed surface area and porosity, using more moderate activation temperature and production time when compared with the physical activation method.^12^ However, the high costs of the chemical agents used in chemical activation can represent a significant disadvantage. One solution to mitigate this problem is to use low-cost activating agents, which can reduce the total cost of the process making it more economically viable.^13^

In this study, the innovative technology for activated carbon production is based on the use of iron-rich materials, particularly ferric oxides, which act as activating agents. In this approach, the iron oxide-rich solid is mixed with carbonized biomass (biochar) and subjected to thermal treatment. During the process, the iron oxide acts as an activating agent and initially undergoes carbothermal reduction, producing carbon monoxide (CO) (eqs 1–3), which also acts as a reducing agent for the iron oxide (eqs 4–6).^14–16^ The CO_2_ generated in reactions (eqs 4–6), in turn, may contribute to the physical activation of carbon at elevated temperatures (eq 7).^14–16^ At the end of the process, activated carbon and metallic iron are obtained, both of which present promising environmental applications.

1 $$3Fe_{2}O_{3}\left(s\right)+C\left(s\right)\to 2Fe_{3}O_{4}\left(s\right)+CO\left(g\right)$$

2 $$Fe_{3}O_{4}\left(s\right)+C\left(s\right)\to 3FeO\left(s\right)+CO\left(g\right)$$

3 FeO(s)+C(s)→Fe(s)+CO(g)

4 $$3Fe_{2}O_{3}\left(s\right)+CO\left(g\right)\to 2Fe_{3}O_{4}\left(s\right)+CO_{2}\left(g\right)$$

5 $$Fe_{3}O_{4}\left(s\right)+CO\left(g\right)\to 3FeO\left(s\right)+CO_{2}\left(g\right)$$

6 $$FeO\left(s\right)+CO\left(g\right)\to Fe\left(s\right)+CO_{2}\left(g\right)$$

7 $$CO_{2}\left(g\right)+C\left(s\right)\to 2CO\left(g\right)$$

To the best of our knowledge, no studies have reported the use of iron oxides or even iron hydroxides as activating agents for activated carbon production. Some studies have employed iron salts or iron oxides to prepare magnetic activated carbons by synthesizing Fe_3_O_4_ or α-FeOOH on the carbon surface;^17–20^ however, none of these studies aimed to use iron oxide as the activating agent itself. Therefore, the novelty of the present process lies in the use of iron oxides as activating agents, distinguishing it from other studies that employ conventional activating agents such as steam, CO_2_, NaOH, KOH, H_3_PO_4_, ZnCl_2_, K_2_CO_3_, FeCl_3_, among others.^21,22^

Thus, our hypothesis is that the use of industrial by-products rich in iron, such as mining tailings and Fe-rich soil samples, can be successfully used as activating agents. For that, coffee bean cake was used as a carbon precursor to obtain novel activated carbons (AC). The materials produced were characterized and applied as adsorbents for pollutant removal in an aqueous system using paracetamol ((N-(4-hydroxyphenyl) acetamide), as defined by the IUPAC nomenclature.^23^ Paracetamol is highly stable in aquatic environments and is not effectively removed by conventional treatments. Despite being present in very low concentrations (in the order of μg/L or ng/L), its presence in water can cause significant chronic effects on the environment and human health, making it necessary to develop efficient methods for its removal.^3,24^ In view of the above, this study seeks new ways of reusing by-products to produce materials that can be applied in contaminant removal processes.

## 2 Methodology

### 2.1 Materials

The coffee bean cake (CC) was provided by the Essential Oils Organic Chemistry Laboratory of the Federal University of Lavras. All reagents used were purchased from Sigma-Aldrich. The Fe-rich soil sample was collected in the region of Bom Sucesso, state of Minas Gerais, Brazil (latitude 21°06′15″, longitude 44°45′20″, altitude 909 m and slope 9°). The sample is classified as a Plinthosol developed over serpentinite and was collected in the A horizon, from 0 to 8 cm deep.^25,26^ The mining waste used was collected in the area affected by the Fundão dam collapse, located in Mariana, belonging to the Metropolitan Mesoregion of Belo Horizonte, Minas Gerais, Brazil.^27^

### 2.2 Coffee Cake Pyrolysis

The coffee bean cake obtained from the coffee extract was initially washed with distilled water and dried at 110 °C in an oven for 12 h. The material was placed into hermetically sealed capsules (without inert gas purging), thus providing a limited O_2_ atmosphere. The CC was then pyrolyzed in a muffle furnace at 450 °C for 90 min, with a heating rate of 20 °C min^–1^ to obtain the pyrolyzed coffee cake (PCC) (Figure S1, Supplementary File). After this process, PCC was treated in a ball mill for 5 min and sieved to obtain a particle size of 150 μm.

### 2.3 Preparation of Activated Carbons

The PCC was activated using innovative activating agents of different origins: commercial (hematite - α-Fe_2_O_3_), natural (Fe-rich soil sample) and residual (Fe-rich mining waste). For this purpose, each activating agent was incorporated separately into PCC at a ratio of 3:1 w/w and then taken to a tubular furnace at 900 °C for 90 min, with a heating rate of 10 °C min^–1^ under N_2_ atmosphere. After obtaining the materials, deionized water was added, followed by sonication for 5 min. The activated carbon was separated from the activating agent magnetically using a neodymium magnet (40 x 20 x 10 mm block) and dried at 60 °C for 12 h. Subsequently, the AC was ground in a ball mill for 5 min. The produced AC samples were then sieved to obtain a particle size of 150 μm. The activated carbons with commercial iron oxide, soil and mining waste were identified as AC_Fe_2_O_3_, AC_soil and AC_waste, respectively.

### 2.4 Characterization

The materials were characterized by infrared spectroscopy with attenuated total reflection (FTIR-ATR) (Varian-660 IR, Pike) in the spectral range of 400–4,000 cm^–1^. Thermogravimetric (TG) analysis was performed under synthetic air and N_2_ flow at a heating rate of 10 °C min^–1^, starting from a temperature of approximately 30 °C up to 900 °C, using Mettler Toledo TG/DSC1 equipment from Shimadzu. Raman spectra were acquired using a WITec Alpha300RA confocal micro-Raman spectrometer equipped with a 633 nm laser. The laser power was set to 2 mW, with an acquisition time of 5 s and 5 accumulations per spectrum. Five different points were analyzed for each sample to ensure spectral reproducibility. The Raman spectra were baseline-corrected using a polynomial fitting procedure in OriginPro. The fitted baseline was subtracted from the original spectra, and the resulting corrected spectra were used to determine the intensities of the D and G bands and calculate the ID/IG ratio. Morphological analyses were performed by scanning electron microscopy (SEM) using LEO EVO 40XVP microscope. The samples were mounted on aluminum stubs using double-sided carbon tape. X-ray diffraction (XRD) analyses were performed using Rigaku equipment, model Ultima IV, with Cu Kα = 1.54051 Å. Scans were performed between angles 15 < 2θ < 80° at a speed of 4 °C min^–1^. Nitrogen adsorption isotherms at 77 K were performed in QuantaChromeAsiQwin 2.0 equipment. Specific surface area and pore distribution analysis were obtained by the BET (Brunauer, Emmett and Teller) and BHJ methods, respectively. Prior to analysis, samples were degassed at 200 °C for 12 h. The methodology of Prahas et al. (2008) with modifications was used to determine the pH at the point of zero charge (pH_PZC_).^29^ For this purpose, 10 mL of 0.1 mol L^–1^ KCl aqueous solution was added under different initial pH conditions (2–11) and 10 mg of material. The pH of the solutions was adjusted with HCl or NaOH. The solutions were mechanically stirred for 24 h and the final pH was measured. The pH at the point of zero charge was determined from the graph of the initial pH versus the final pH. The mining waste sample was analyzed by inductively coupled plasma optical emission spectrometry (ICP-OES) after digestion according to the USEPA 3051A method. In this procedure, the extracting solution consisted of a 3:1 mixture of nitric and hydrochloric acids, applied under high temperature and pressure in a microwave digestion system. After digestion, the samples were filtered using quantitative filter paper, rinsed with ultrapure water, transferred to 50.0 mL volumetric flasks, and the volume was completed with ultrapure water. The digestion of the chars AC_Fe_2_O_3_, AC_soil and AC_waste was performed according to the methodology proposed by Enders & Lehmann (2012), with modifications. The ICP-OES analysis was conducted using a SPECTRO SPECTROBLUE ICP-OES instrument. An RF power of 1400 W was applied, with an argon gas flow rate of 12 L min^–1^, while the auxiliary and nebulizer gas flow rates were set at 0.80 L min^–1^ and 0.85 L.

### 2.5 Adsorption Experiments

The adsorption kinetics of the samples CC, PCC, AC_Fe_2_O_3_, AC_soil and AC_waste were performed by adding 10 mg of sample to 10 mL of paracetamol solution at a concentration of 10 mg L^–1^. The system was left under magnetic stirring at 25 ± 2 °C and an aliquot of the mixture was centrifuged (5 min at 3000 rpm) and the supernatant was collected at different time intervals. The absorbance was quantified by UV-visible spectroscopy (λ_max_ = 243 nm). The analytical curve of paracetamol was used to obtain the residual concentrations. The adsorption capacity was evaluated by the amount of material adsorbed per mass of adsorbent (q_e_) as a function of the adsorbate concentration at equilibrium in the solution (C_e_) (eq 8), in which C_0_ represents the initial adsorbate concentration.

8 $$q_{e}\left(mgg^{-1}\right)=\frac{C_{0}-C_{e}}{m}\times V$$

The adsorption isotherms were obtained by adding 10 mg of material to 10 mL of paracetamol solution at different concentrations (2.5, 5, 10, 25, 50, 100, 250, 500 mg L^–1^). The mixture was kept under magnetic stirring at a temperature of 25 ± 2 °C. After 24 h of contact, the supernatant was separated by centrifugation, analyzed by UV-vis spectroscopy at λ_max_ = 243 nm, and the residual adsorbate concentration was obtained using the analytical curve.

## 3 Results and Discussion

### 3.1 Pyrolysis and Activation

Carbonization or pyrolysis is the thermochemical decomposition of raw materials under an inert or reduced oxygen atmosphere, with the aim of removing non-carbonaceous species such as nitrogen, oxygen and hydrogen and increasing the fixed carbon content to produce biochar.^12^ In the pyrolysis process, both the raw material source and the experimental parameters can significantly affect the physicochemical properties and yield of the final products. Regarding the experimental parameters, temperature has the most significant effect. The pyrolysis temperature range mostly mentioned in the literature is 300–900 °C.^30^ In this range, the treatment results in the loss of water, carbon dioxide and a wide range of organic molecules, such as alcohols, ketones and acids.^12^ Generally, increasing pyrolysis temperature is associated with higher biochar quality, but with lower yields.^31,32^ In this context, the structural composition of plant biomass is the main indicator of thermal stability. Typically, biomasses with higher lignin contents demonstrate greater thermal stability, which results in a higher biochar yield.^33^ Coffee residues usually contain about 10–13.2%, 30–40%, and 19.8–29.8% of cellulose, hemicellulose, and lignin, respectively.^34,35^

Figure 1 shows the thermal decomposition profile of CC in an inert and oxidizing atmosphere analyzed by thermogravimetry. In an oxidizing atmosphere, four distinct thermal events can be observed: the first is attributed to the elimination of water adsorbed on the surface of the material, resulting in a weight loss of approximately 8.35%, at a temperature below 150 °C. The second (42.2%) and third (9.71%) weight losses, which occur between 205 and 330 °C and 330 and 408 °C, respectively, are the result of the depolymerization of cellulose and hemicellulose into oligosaccharides, and of other volatile components present in the coffee cake. The fourth and last thermal event begins close to 408 °C and extends to 511 °C, with a weight loss of 28.9%. This loss can be mainly attributed to the thermal decomposition of lignin, which is the most thermally stable among the lignocellulosic components of coffee cake.^36–38^ After heat treatment at 900 °C, the final residue corresponds to 8.6% of the original mass, representing the mineral fraction of CC.

**Figure 1:**
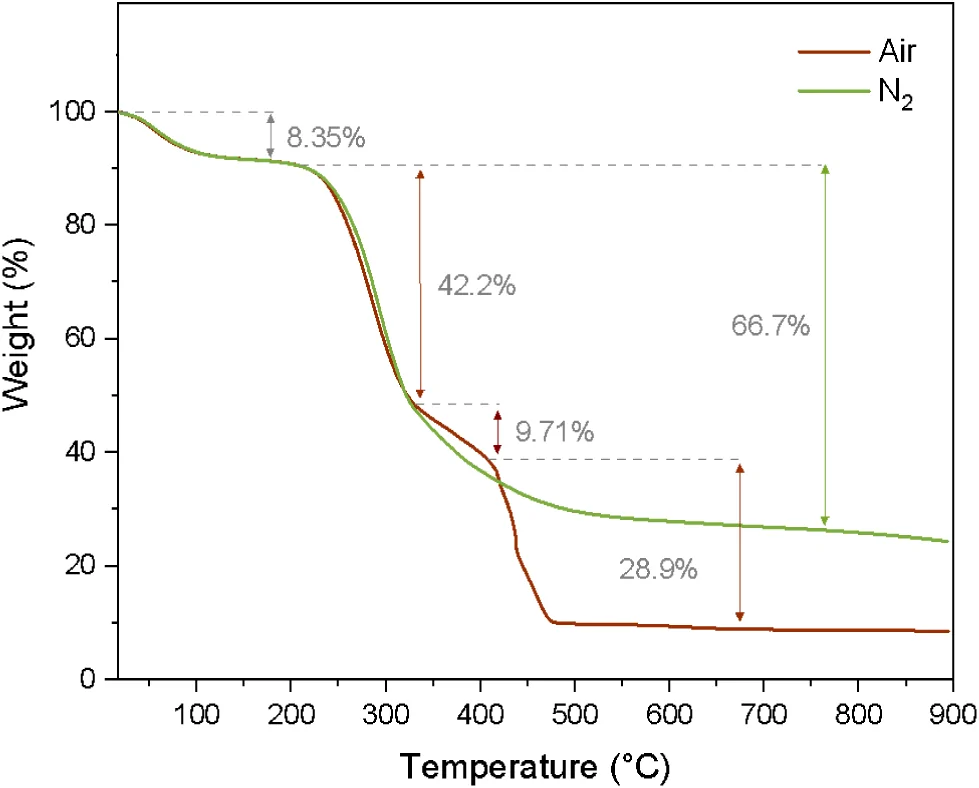
TGA curve of CC in an atmosphere under synthetic air and N_2_ flow (10 °C min^–1^).

Under an inert atmosphere, two thermal events accompanied by weight loss are observed. The first event is attributed to the elimination of water adsorbed on the surface of the material, resulting in a mass loss of approximately 8.35%. The second and largest weight loss of 66.7% occurs due to the depolymerization of cellulose, hemicellulose and lignin. This second weight loss extends from 200 °C to 800 °C. This decomposition profile can be attributed to the thermal decomposition characteristics of lignin. At the end of the thermal process at 900 °C, a final residue of approximately 24.4% is observed. Up to 8.6% corresponds to the mineral composition of the coffee cake, according to the results of the thermogravimetric analysis in an oxidizing atmosphere. The amount of fixed carbon calculated was 15.8%.

Based on these results, the pyrolysis temperature of 450 °C was chosen, as it proved to be effective in removing the volatile fraction of the CC residue. After pyrolysis performed in a muffle furnace, the yield of the PCC was 30.1%. The fixed carbon content was 22.1%. As for the PCC activation process, the yields of the materials AC_Fe_2_O_3_, AC_soil and AC_waste were 13.5%, 13.5% and 8.6%, respectively.

According to eqs 1 to 7, the activation process involves reactions involving the reduction of iron oxides to metallic iron and consequently oxidation of the carbon present in the coal, which results in the formation of the pores present in the AC. In this context, X-ray diffractometry analysis of the activating agents was performed before and after the activation process (Figure 2). The purpose was to identify possible modifications in the iron oxide phases resulting from changes in their oxidation state. The phases were identified by comparison with the crystalline pattern of solids consulted in the QualX 2.0 database.^39^ The comparison between the X-ray diffractograms of the samples and the reference charts can be found in Figure S2 of the Supplementary Material.

**Figure 2:**
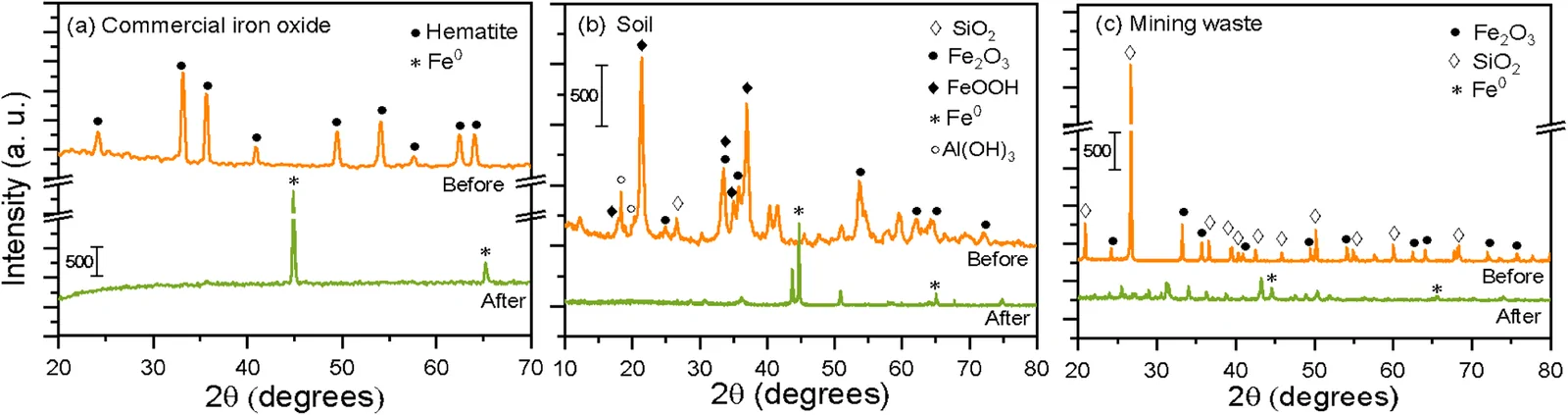
X-ray diffractograms obtained for the samples (a) commercial iron oxide (b) soil and (c) mining residue before and after the activation process.

In the diffractogram of the commercial iron oxide (Figure 2a), the reagent is composed exclusively of the hematite phase (α-Fe_2_O_3_). After activation, the disappearance of the signals related to hematite and the presence of diffraction lines characteristic of metallic iron are observed, confirming that Fe^3+^ was reduced to Fe^0^, according to eqs 1–6. The diffractograms of the soil sample (Figure 2b) show a heterogeneous profile composed mainly of hematite (α-Fe_2_O_3_) and goethite (α-FeOOH), in addition to silicon dioxide (SiO_2_) and gibbsite (Al­(OH)_3_). Araujo et al. (2014) identified these same phases when studying the mineralogy of this soil, and the chemical composition obtained was 72.33, 20.98, 12.91, and 2.53% of Fe_2_O_3_, Al_2_O_3_, SiO_2_, and MgO, respectively, as the major phases.^26^ After activation, diffraction signals at 2θ equal to 45° and 65° were observed, corresponding to metallic iron. The low-intensity signal at 36.1° suggests the presence of magnetite (Fe_3_O_4_). In the XRD pattern of the mining waste after thermal treatment, the appearance of diffraction lines characteristic of Fe^0^ (45° and 65°) can also be observed, however with lower intensity (Figure 2c). This result is related to the lower iron content in the mining waste sample, as indicated by the ICP-OES results, in which the following contents of Fe_2_O_3_, SiO_2_, and Al_2_O_3_ were obtained: 32.7, 54.4, and 26.1%, respectively. Similar results were reported by Ribeiro et al. (2023), in which 148 samples of iron-rich tailings and soil were collected along a 47 km stretch of the Gualaxo do Norte River, which was severely contaminated by tailings released after the collapse of the Fundão dam in Mariana, Minas Gerais, Brazil.^27^ These results confirm that during the activation process carbothermic reduction of the oxidized iron phases present in the samples used as activating agents occurred. XRD results reported by Abanades and d’Izard (2025) also confirmed the formation of Fe^0^ when biochar was used to produce metallic iron through carbothermal reduction.^16^ The weakening or disappearance of the SiO_2_ and Al­(OH)_3_ peaks in the Figure 2b and 2c is attributed to the post-treatment magnetic separation step. This behavior suggests that these mineral phases are no longer present in the isolated solid residue in detectable amounts by XRD. This observation can be explained by the redistribution of Si- and Al-containing species during the activation process, leading to their incorporation into the activated carbon fraction.^28,69^

### 3.2 Characterization of Activated Carbons

The morphology of PCC (before sieving using a 100 Mesh sieve) and activated carbons (AC_Fe_2_O_3_, AC_soil, and AC_waste) was evaluated using scanning electron microscopy (SEM - Figure 3). In the PCC image (Figure 3a), irregular structures of varying sizes are observed, with the presence of cavities characteristic of lignocellulosic materials.^40^ Upon magnification of this image (Figure 3b and 3c), the structures become clearly visible, with the bright contrast highlighting the cavity walls and the darker contrast emphasizing the interiors. At higher magnification (Figure 3c), the PCC particles exhibit a smoother and more uniform surface morphology. The images related to the activated carbons are quite similar (Figure 3d–i). A reduction in particle size is observed due to the sieving of the precursor, along with the presence of more regular structures, smooth surfaces and particles with a broad size distribution.

**Figure 3:**
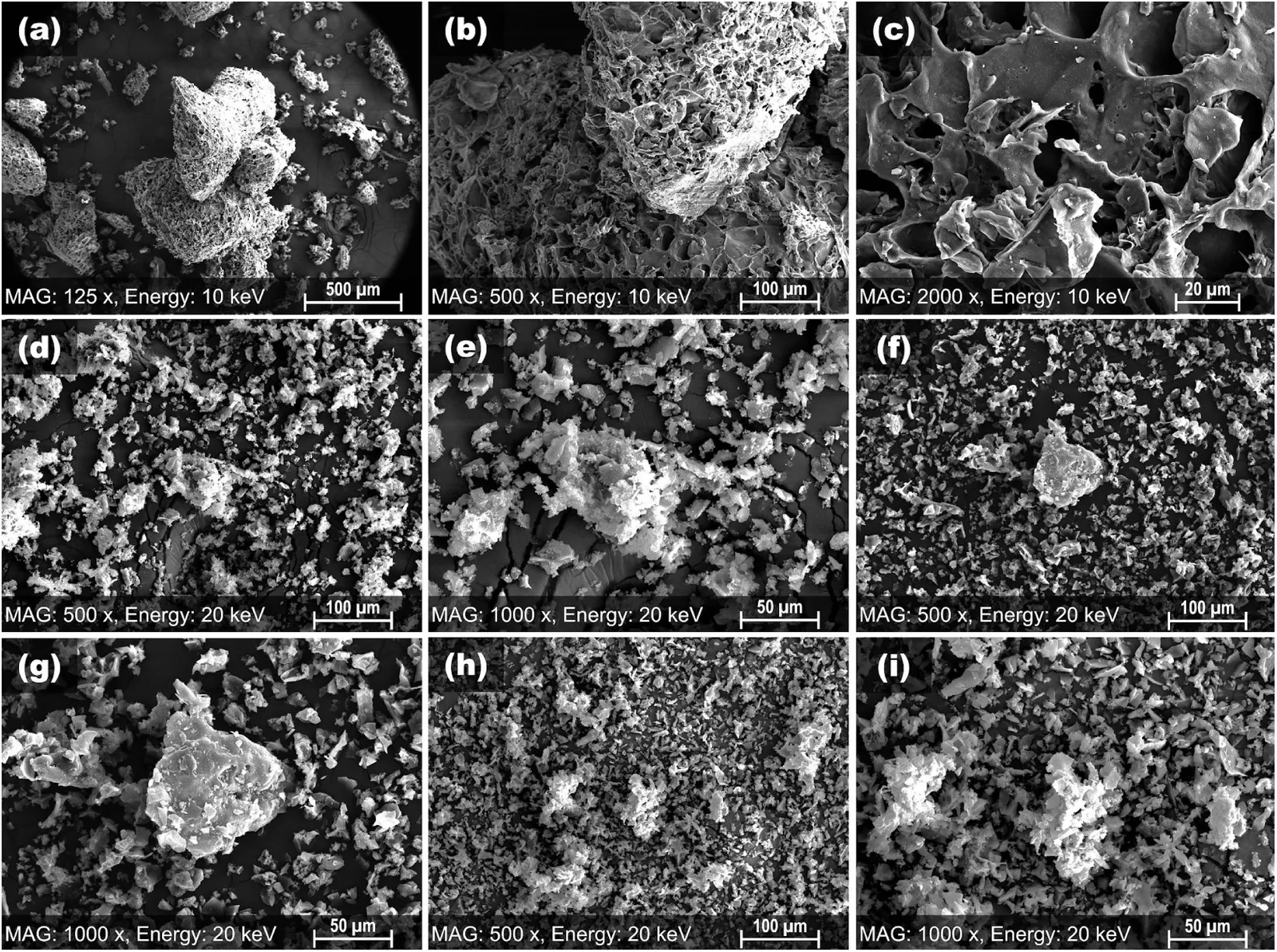
SEM images for the samples: (a–c) PCC; (d, e) AC_Fe_2_O_3_; (f, g) AC_soil; (h, i) AC_waste, obtained at different magnifications.

The activated carbons were prepared using activating agents composed of one or more mineral phases. Due to the presence of heavier elements such as aluminum, silicon, and iron, identified by XRD analysis, these phases exhibit a brighter contrast compared to the carbon phase, as shown in the elemental mapping images in Figure 4a–i. EDS analysis, in addition to providing the chemical composition of the materials, allows for an understanding of the distribution of these mineral phases in carbon-rich materials by using compositional contrast. Table 1 presents the semi-quantitative amounts of elements, excluding carbon, present in the samples, as determined by EDS analysis. The results for the activated carbons AC_Fe_2_O_3_, AC_soil, and AC_waste reveal a high amount of residual iron in the samples. Among them, the highest iron content was detected in AC_Fe_2_O_3_, followed by AC_soil and AC_waste. These results are consistent with elemental analysis conducted by ICP-OES, which revealed approximately 14.8, 9.70, and 6.50% iron in the AC_Fe_2_O_3_, AC_soil, and AC_waste samples, respectively. In addition to iron, other major elements quantified were oxygen, aluminum, and silicon. The elemental mapping of the activated carbons shows that the elements, especially iron, are distributed almost uniformly across the surface of the carbons. The detection of Al and Si in the final activated carbon fraction indicates that Al- and Si-containing phases were incorporated into or remained associated with the carbonaceous matrix. This observation reinforces the interpretation derived from the XRD results, confirming that these mineral species are predominantly retained in the activated carbon fraction rather than the separated residue.

**Figure 4:**
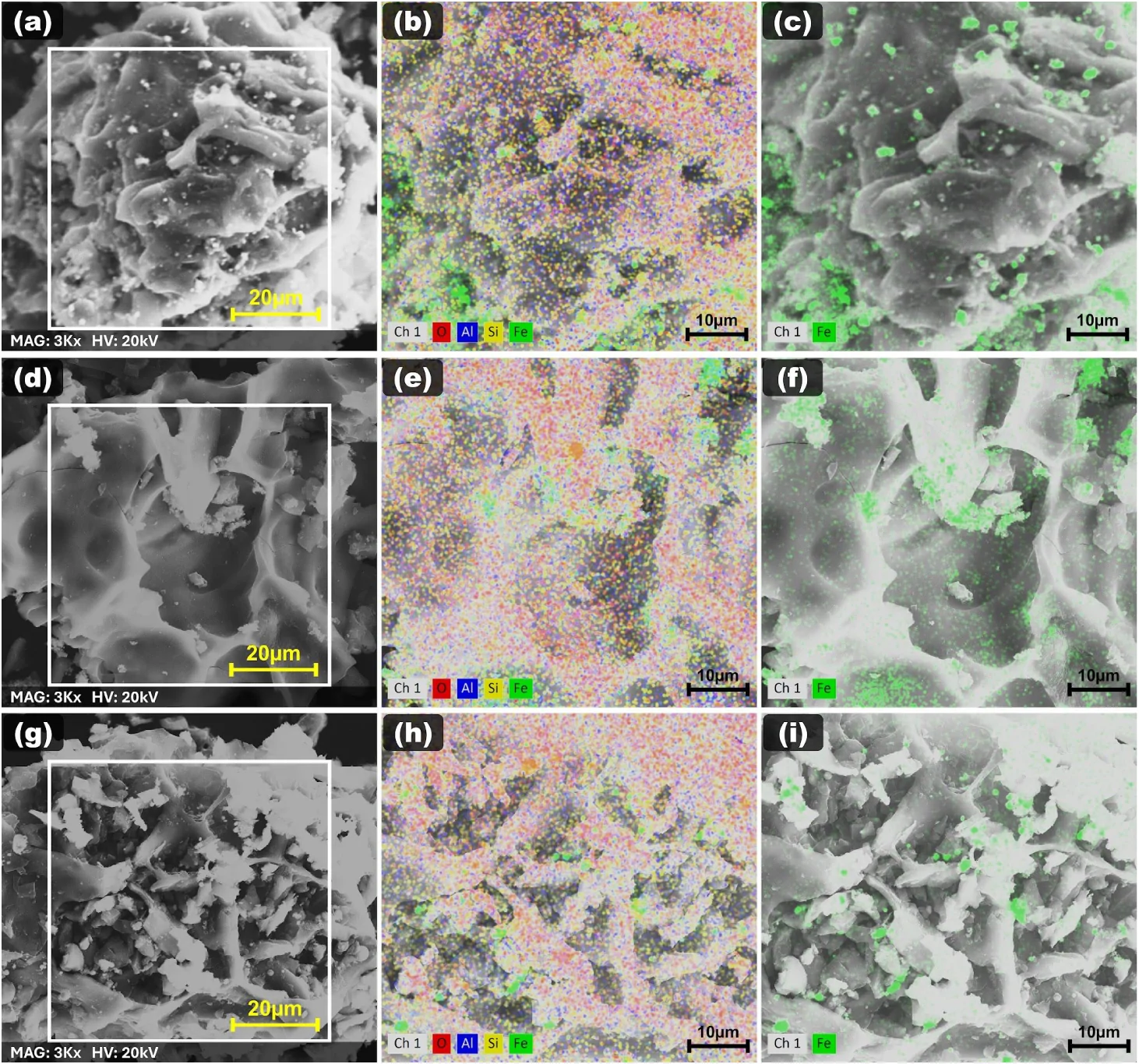
EDS mapping of the samples: (a–c) AC_Fe_2_O_3_; (d–f) AC_soil; (g–i) AC_waste.

**Table 1:** Semi-Quantitative EDS Analysis of the Samples AC_Fe_2_O_3_, AC_soil, and AC_waste

	Normalized mass of elements (%)			
Materials	Fe	O	Si	Al
AC_Fe_2_O_3_	45.9	42.2	4.8	3.1
AC_soil	32.4	41.2	8.1	12.8
AC_waste	27.6	49.1	12.3	7.5

Analysis by infrared spectroscopy with attenuated total reflection (FTIR-ATR) was conducted to evaluate the functional groups present in the materials, and the results are shown in Figure 5. The spectrum of the CC (Figure 5a) shows a band at 3300 cm^–1^, which can be attributed to the O–H vibrations characteristic of phenolic functional groups, as well as hydroxyls of the cellulose and hemicellulose structure present in the coffee beans. Moreover, it can be related to the presence of water adsorbed on the surface of the material. At 2930 cm^–1^ and 2840 cm^–1^, two bands referring to C–H vibrations of methyl and methylene groups are observed. The absorption between 1749–1621 cm^–1^ is characteristic of C=O vibrational stretching of carbonyl. This axial deformation vibration of the C–O bond suggests the presence of both carboxylic acids and esters or δ-lactones linked to aromatic groups.^41^ In regions of lower frequencies, absorptions characteristic of angular deformation of the C–H and C–O bonds of alcohols are identified at 1375 cm^–1^ and 1229 cm^–1^, respectively. The absorption bands of C–O–C bonds represent the vibration of the pyranose ring at 1050 cm^–1^ and the β-glycosidic bonds between glycan units at 903 cm^–1^, which are characteristic of cellulose and hemicellulose.

**Figure 5:**
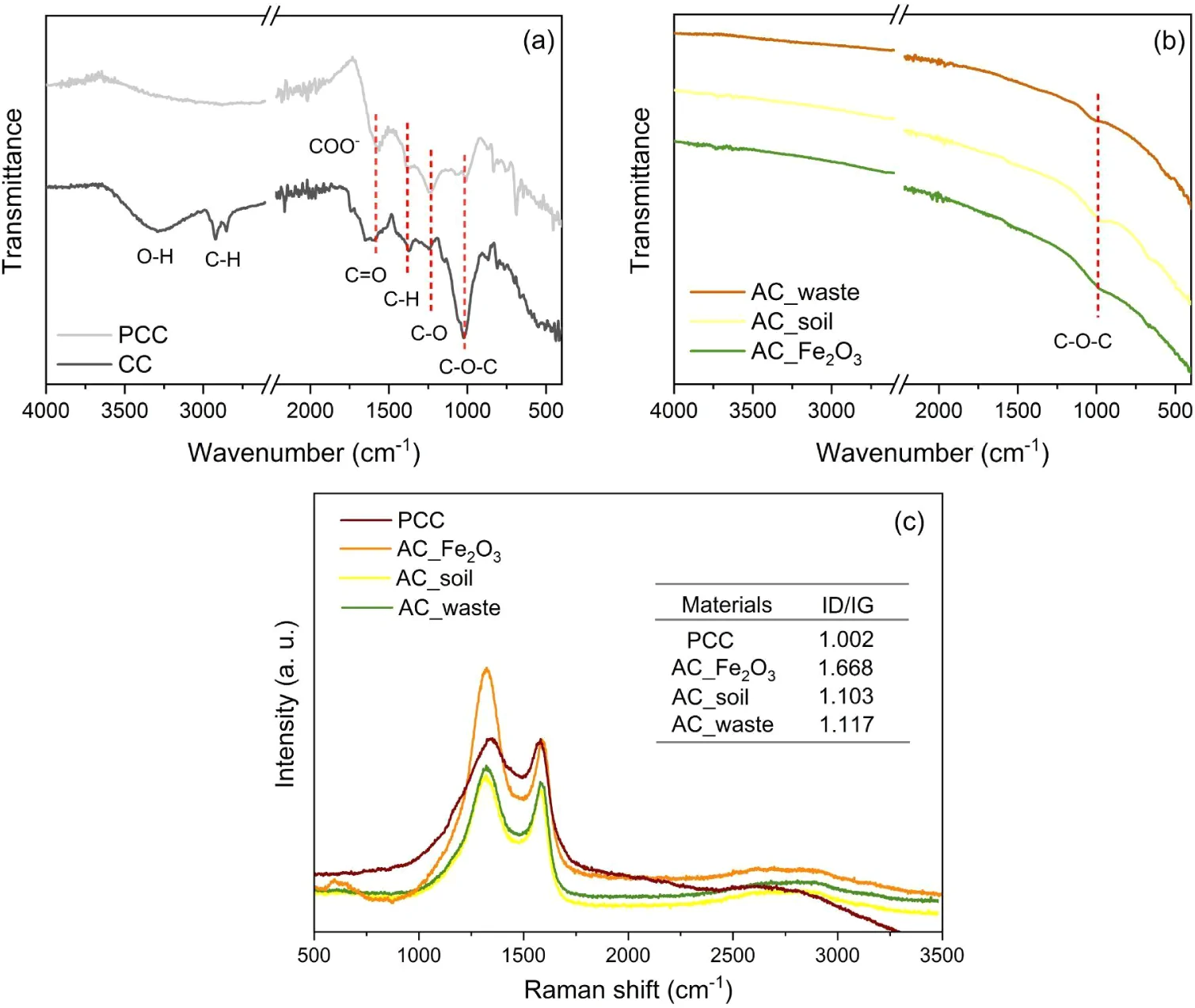
Attenuated total reflectance Fourier transform infrared (FTIR-ATR) spectra of (a) CC and PCC; (b) activated carbons: AC_Fe_2_O_3_, AC_soil and AC_waste; and (c) Raman spectra of PCC, AC_Fe_2_O_3_, AC_soil, and AC_waste recorded using a 633 nm laser. Baseline correction was applied prior to the calculation of the ID/IG ratio.

After the pyrolysis process there was a reduction in the intensity or disappearance of the transmittance bands of the PCC sample in relation to CC (Figure 5a). The most evident losses were of the bands at 2930 cm^–1^ and 2840 cm^–1^ attributed to vibrational stretching of C–H groups, the band at 3300 cm^–1^ attributed to O–H vibrations and the band at 1050 cm^–1^ related to vibrations of C–O–C bonds. On the other hand, the band at 1590 cm^–1^ became more evident. This band is related to the conversion of carboxylic groups to carboxylate ions present in biochar structures.^40,42^ The FTIR analysis performed after the activation process of PCC showed the disappearance of the transmittance bands (Figure 5b) and very similar spectral profiles for the activated carbons (AC_Fe_2_O_3_; AC_soil and AC_waste). A single band close to 1000 cm^–1^ was identified in the spectra of the activated carbons and was attributed to the C–O–C vibrations.

The Raman spectra of the PCC, AC_Fe_2_O_3_, AC_soil, and AC_waste samples (Figure 5c) exhibited two characteristic bands of carbonaceous materials: the D band, located at approximately 1340–1360 cm^–1^, and the G band, centered around 1580–1600 cm^–1^. The D band is associated with structural defects, crystallite edges, and disordered carbon, whereas the G band corresponds to the stretching vibrations of sp^2^ -hybridized carbon atoms in graphitic structures.^43^

The ID/IG ratio was used to compare the degree of structural disorder among the samples. The carbonized precursor (PCC) exhibited an ID/IG ratio of 1.002. After activation, an increase in this ratio was observed for all samples, indicating that the activation process modified the carbon structure and increased the density of structural defects. Among the activated materials, the AC_Fe_2_O_3_ sample showed the highest ID/IG value (1.668), which was significantly higher than those of the carbonized precursor and the other activated carbons. This result indicates that the use of commercial α-Fe_2_O_3_ promoted greater disruption of the carbon framework during the thermal treatment, favoring the formation of structural defects and reducing the average size of the graphitic domains.

In contrast, the AC_soil (ID/IG= 1.103) and AC_waste (ID/IG= 1.117) samples exhibited only a slight increase in the ID/IG ratio compared with PCC. This result suggests that commercial α-Fe_2_O_3_ promoted a greater generation of structural defects in the carbon during activation, possibly due to the higher availability of iron species compared with the complex mineral sources. However, the soil and mining waste samples also contain iron, in addition to other mineral constituents, such as Si, Al, and Mg oxides, as well as other elements, as identified by XRD (Figure 2b and 2c). Therefore, the lack of normalization of the iron concentration among the different activating agents prevents the isolated attribution of the observed structural defects solely to the effect of iron.

The thermogravimetric analysis (TGA) in an oxidizing atmosphere of the PCC and activated carbons is illustrated in Figure 6. The PCC curve revealed three main mass loss regions (Figure 6a). The first loss, of 12.6%, occurred below 200 °C, corresponding to the elimination of moisture from the sample. The second mass loss, amounting to 24.2%, was accompanied by a slight exothermic signal, with a maximum temperature at 404 °C, attributed to the decomposition and volatilization of oxygenated functional groups present in the PCC. This interpretation is supported by the FTIR analysis (Figure 5a), which indicated the presence of bands associated with C–O, C=O, O=C–O, and C–O–C bonds in the PCC. The most significant mass loss (51.9%) occurred in the temperature range of 423 °C to 500 °C, accompanied by an intense exothermic signal, with maximum intensity on the DTA curve at 440 °C. This loss corresponds to carbon oxidation, which continues, though less pronounced, up to 900 °C with a mass loss of 5.3%. The final residue, consisting of the mineral fraction of the PCC, is approximately 11%.

**Figure 6:**
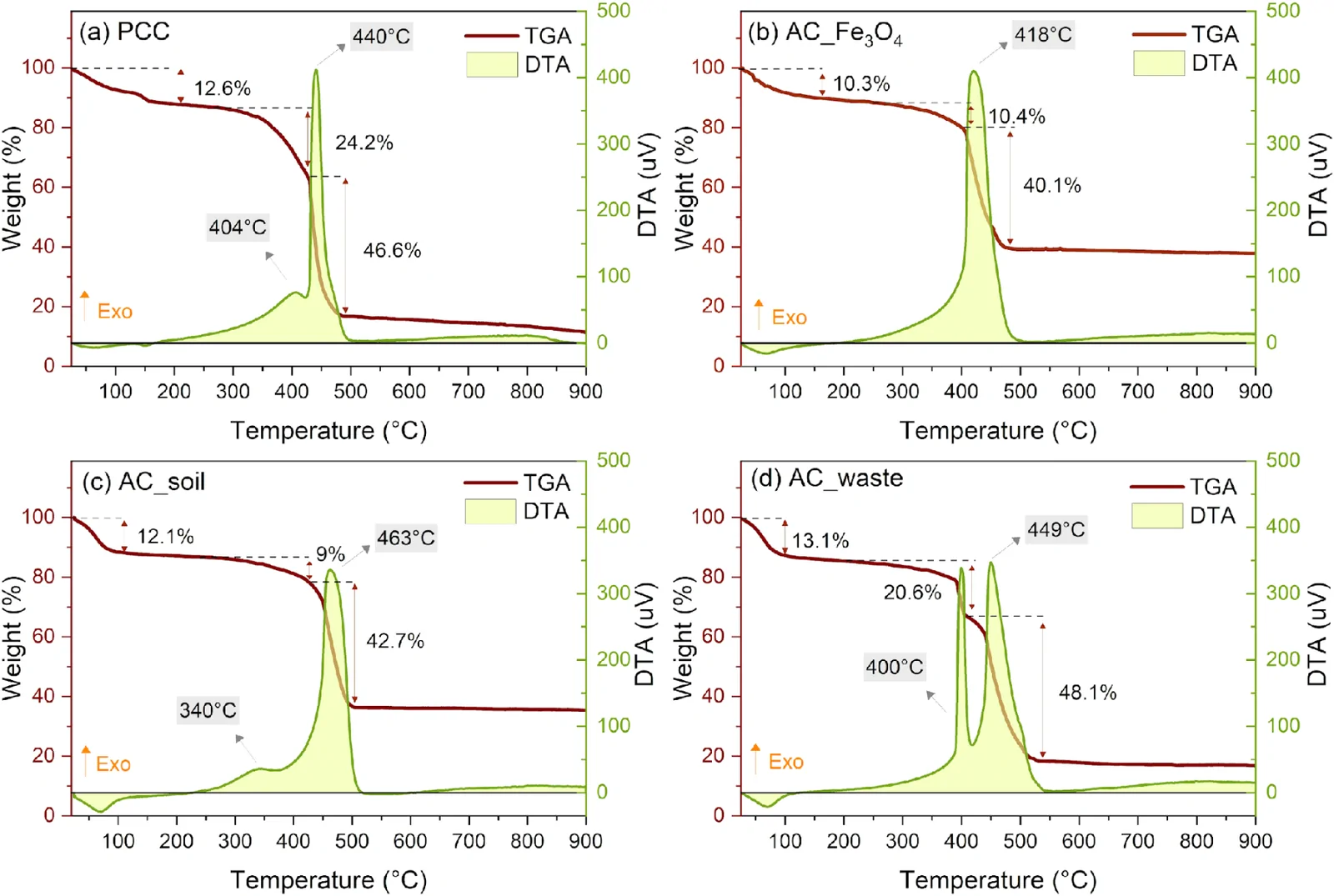
TGA and DTA curves of the samples (a) PCC, (b) AC_Fe_2_O_3_, (c) AC_soil, and (d) AC_waste in an air flow atmosphere (10 °C min^–1^).

The activated carbon samples AC_Fe_3_O_4_, AC_soil, and AC_waste exhibited three distinct stages of mass loss, as shown in Figure 6b, 6c, and 6d. The first mass loss, accompanied by an endothermic signal below 150 °C, was attributed to the dehydration of the samples. The mass loss values were 10.3%, 12.1%, and 13.1% for the AC_Fe_2_O_3_, AC_soil, and AC_waste samples, respectively. The second mass loss of the activated carbons occurred progressively, without a clearly identifiable maximum loss temperature through the exothermic signal on the DTA curve for the AC_Fe_2_O_3_ sample. On the other hand, in Figure 6c and 6d (for AC_soil and AC_waste samples, respectively), the maximum mass loss temperature can be identified, being more evident for AC_waste, which showed an intense exothermic signal at 400 °C. AC_Fe_2_O_3_ showed a loss of 10.4%, AC_soil a loss of 9%, and AC_waste a loss of 20.6%. This loss may be related to functional groups present on the surface of the carbons, responsible for the small transmittance band around 1000 cm^–1^ in the FTIR spectra (Figure 5b). The third and final mass loss, of 40.1% for AC_Fe_2_O_3_, 42.7% for AC_soil, and 48.1% for AC_waste, occurred with maximum loss peaks at 418 °C, 463 °C, and 449 °C, respectively, as identified by the intense exothermic signal on the DTA curve. The final residue contents for the AC_Fe_2_O_3_, AC_soil, and AC_waste samples were approximately 37.9%, 35.5%, and 18.2%, respectively. Considering that 11% corresponds to the ash content of the PCC, the remaining 26.9%, 24.5%, and 7.2% can be attributed to the mineral fraction from the raw materials used (AC_Fe_2_O_3_, soil, and waste) to obtain the AC_Fe_2_O_3_, AC_soil, and AC_waste samples, respectively.

The N_2_ adsorption isotherms at 77 K and pore distribution curves of the AC and PCC samples are shown in Figure 7. The difference in the volume of adsorbed N_2_ between the PCC control sample and AC_Fe_2_O_3_, AC_soil and AC_waste is notable, which is also reflected in the adsorption profile of these materials. The PCC isotherm indicates a low volume of adsorbed N_2_ at low relative pressures, gradually increasing as p/p_0_ increases and remaining finite at saturation pressure (p/p_0_ = 1). The physisorption isotherm profile resembles Type III, as defined by IUPAC.^43^ It indicates relatively weak adsorbent-adsorbate interactions and clustering of adsorbed molecules around the most favorable sites on the surface of a non-porous or macroporous solid, with a size greater than 50 nm.^44^ Sample AC_Fe_2_O_3_ showed the highest volume of adsorbed N_2_ at low relative pressure among the materials. The isotherm was similar to type IV­(a) characteristic of mesoporous solids.^44,45^ The samples AC_soil and AC_waste also showed Type IV­(a) isotherms. However, they showed lower adsorption at low relative pressure when compared with AC_Fe_2_O_3_. Furthermore, it is possible to distinguish in the isotherms of these samples the relative pressure corresponding to the end of the monolayer coverage through the sharper inflection point at p/p_0_ < 0.1 when compared with AC_Fe_2_O_3_, which has a more gradual inflection point, making its identification difficult. The quasi-linear region that follows the inflection point in the isotherms of AC_soil and AC_waste is attributed to the onset of multilayer adsorption, which in the isotherm of AC_Fe_2_O_3_ is confused with significant overlapping of the monolayer coverage. In the isotherms of the three activated carbons, AC_Fe_2_O_3_, AC_soil and AC_waste, capillary condensation is accompanied by hysteresis. This occurs when the mesopore width exceeds the critical width of approximately 4 nm. This fact is confirmed by the pore size distribution curves (Figure 7b), which indicate the presence of mesopores with sizes in the range of approximately 3–5 nm. The shape of the hysteresis in the isotherms of the activated carbons is similar to Type H3 hysteresis, which corresponds to non-rigid aggregates of plate-shaped particles.

**Figure 7:**
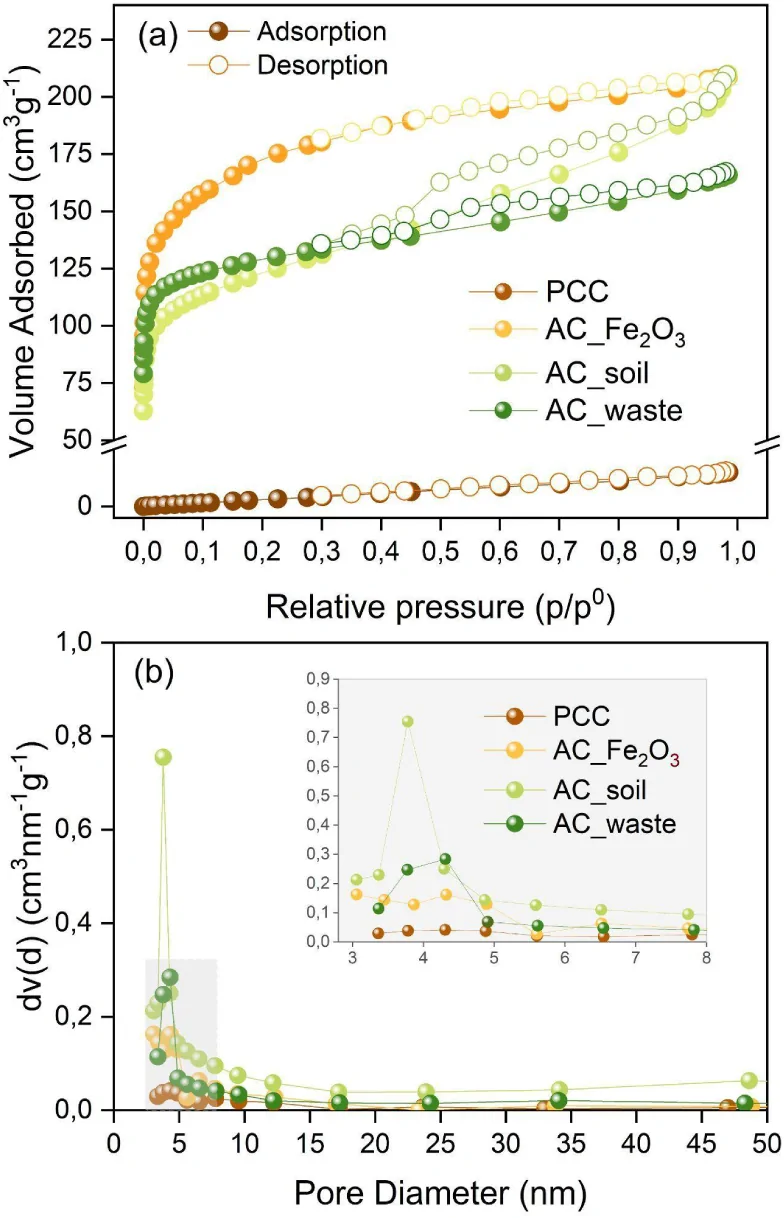
(a) N_2_ adsorption/desorption isotherms at 77 K; (b) pore size distribution obtained from the desorption branches of the N_2_ isotherms using the BJH method (image within Figure 4b represents the highlighted region).

The biochar prepared from coffee cake (sample PCC) showed a low BET surface area (*S*_BET_) of 21 m^2^ g^–1^. This result was expected due to the biochar production process, which involves the pyrolysis of biomass at moderate temperatures (400–700°C). Such conditions generally result in materials with lower porosity and specific surface area, which is in accordance with the surface area values for most biochars reported in the literature.^13^ Moreover, the pore structure may be obstructed by the deposition of volatile substances formed during pyrolysis, which may further reduce the surface area and adsorption capacity of these materials. These substances can be removed by activating the material at higher temperatures using a suitable activation agent.^46^ The activation of PCC with commercial hematite, soil and mining waste samples promoted an increase of 29, 27, and 22 times, respectively, in the BET surface area when compared with the biochar. Among the materials, the activated carbon prepared using commercial hematite (AC_Fe_2_O_3_) had a larger surface area (614 m^2^ g^–1^) when compared with AC_soil (578 m^2^ g^–1^) and AC_waste (458 m^2^ g^–1^). The smaller surface area obtained for AC_soil and AC_waste may be related both to the amount of iron present in the soil (50.6% w/w) and mining waste (22.9% w/w) samples and to the iron phase that constitutes them. As previously mentioned, for the activation process to effectively occur, it is important that iron is present in its oxidized form (Fe^3+^), since the activation process involves the donation of electrons from the coal to the iron, creating active sites. The AC samples AC_Fe_2_O_3_, AC_soil, and AC_waste were prepared using the same activation agent, however, obtained from different sources. Therefore, it is important to correlate their properties with the amount of the activating agent itself (Fe^3+^) available in the commercial hematite, soil, and mining waste samples. Since commercial hematite has a higher content of Fe^3+^ (69.9% w/w), there is a greater availability of this species for the activation process to occur. In contrast, the soil and waste samples are characterized by a heterogeneous composition, with high amounts of other elements, such as Al, Si, and O, as determined by XRD analysis (Figure 2). In this study, the amounts of soil and mining waste used in the activation of the pyrolyzed coffee cake were not adjusted to match the amount of Fe^3+^ present in the commercial iron oxide. This may have compromised the activation process of the AC_soil and AC_waste materials. Even so, the *S*_BET_ values obtained are similar to those reported in the literature for activated carbons.^13^ Overall, these results confirm the efficiency of the α-Fe_2_O_3_ present in the studied samples in the carbon activation process.

The pH at the point of zero charge (pH_PZC_) analysis was performed to verify the net surface charges of the materials, resulting from their acidic or basic characteristics. The results obtained are shown in Figure 8, with value ranging from 5.4 to 7.5. The lowest pH_PZC_ value for CC of 5.4 is attributed to the presence of oxygenated functional groups in the biomass structure, such as carboxylic, lactonic and phenolic groups, which were removed after pyrolysis. This resulted in an increase in the pH_PZC_ value (7.5) for PCC. In the literature, pH_PZC_ values between 3.0–12.0 have been reported for biochars.^40^ The variation of these values is dependent on the pyrolysis conditions, such as time and temperature. Generally, an increase in pyrolysis temperature and time leads to an increase in the pH_PZC_ value.^40^ This occurs because more severe pyrolysis conditions tend to modify the surface of the material, increasing the amount of basic sites due to dehydroxylation and decomposition of acidic functional groups, which leads to greater basicity of the surface of the material.^47,48^ The pH_PZC_ values for the activated carbons were lower than the pH_PZC_ value of the PCC sample. It is noted, however, that AC_Fe_2_O_3_ showed a pH_PZC_ value very similar to PCC, while AC_soil and AC_waste showed lower pH_PZC_ values, indicating a slightly acidic character.

**Figure 8:**
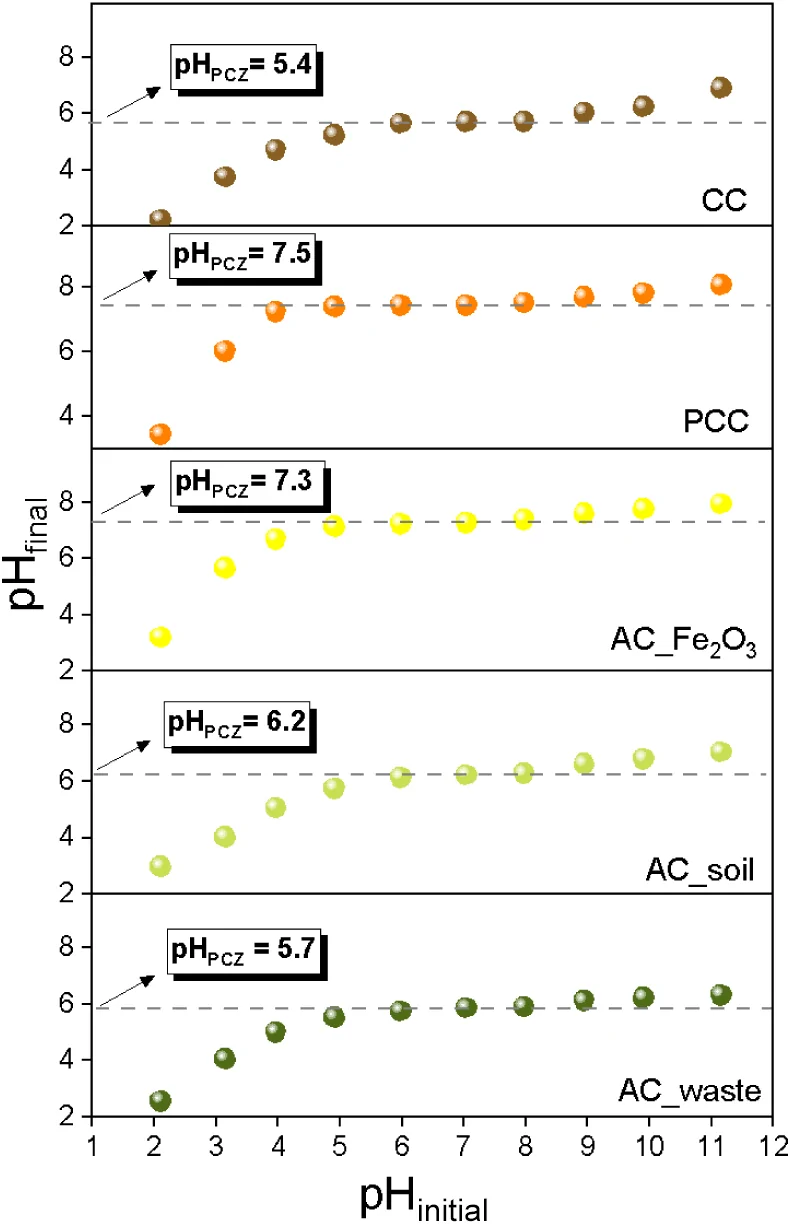
pH at the point of zero charge (pHPZC) of the samples CC, PCC, AC_Fe2O3, AC_soil and AC_waste.

### 3.3 Adsorption

The activated carbons were used in the adsorption of paracetamol and the results of the adsorption kinetics are shown in Figure 9. In general, the adsorption of paracetamol was faster in the first minutes of contact, with the kinetics decreasing until reaching equilibrium close to 180 min. The removal percentages for AC_Fe_2_O_3_, AC_soil and AC_waste demonstrated excellent capacities for removing the drug from an aqueous medium, with values equal to 82.5%, 67.1% and 51.4%, respectively. On the other hand, the control materials CC and PCC adsorbed only 4.2% and 3.7%, respectively.

**Figure 9:**
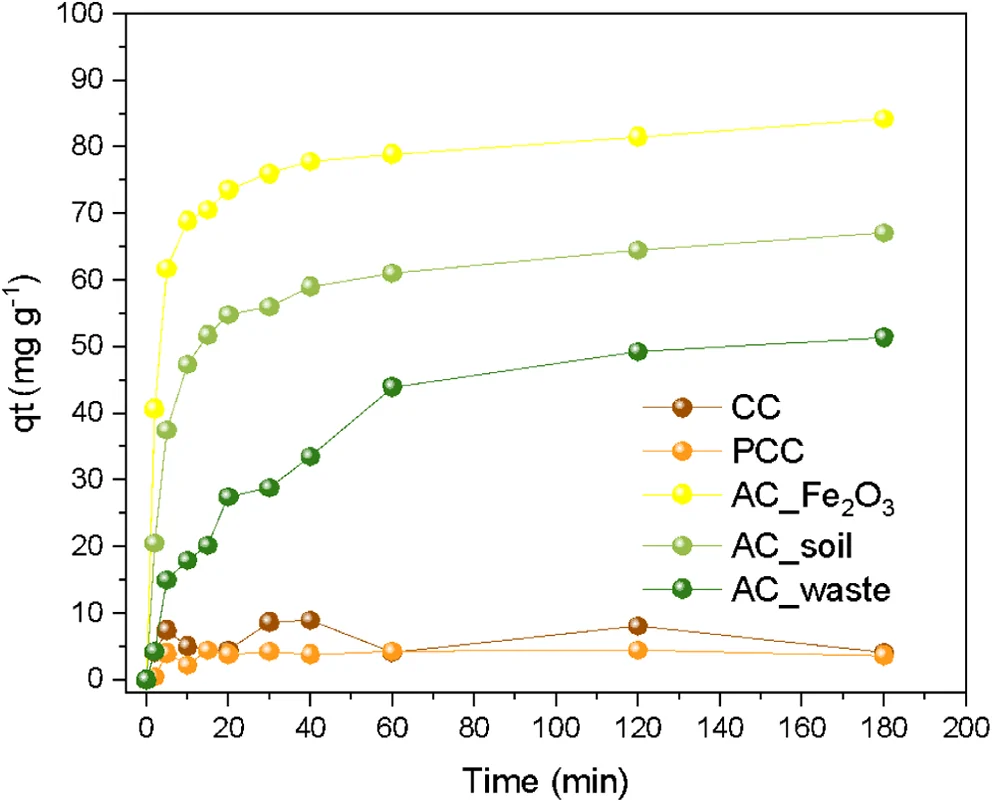
Adsorption kinetics of paracetamol (10 mg L^–1^) onto samples CC, PCC, AC_Fe_2_O_3_, AC_soil and AC_waste (1 g L^–1^) at pH 5.6.

The adsorption process on activated carbons can be influenced by several factors. The main ones include the interaction between adsorbent and adsorbate, the pH of the solution, the pH at the point of zero charge (pHpzc), the porosity and the surface area of the adsorbent.^49^ The interactions between the adsorbent and the adsorbate depend on the electrostatic forces, non-electrostatic forces and dispersive forces between them.^50^ Although pH_PZC_ values provide useful information regarding the surface charge characteristics of the activated carbons, the absence of adsorption experiments under different pH conditions does not allow a definitive assessment of the contribution of electrostatic interactions to the adsorption mechanism. In this context, knowledge about the surface charge of the adsorbent and adsorbate allows predicting the forces of attraction or repulsion between them, so that the pH value may or may not favor the adsorptive process.^29^ According to the pH_PZC_ values of the activated carbons shown in Figure 8, in solutions with pH < pH_PZC_, the surface of the activated carbon has a global positive charge and the adsorption of anionic pollutants will be favored. In solutions with pH > pH_PZC_, the surface of the activated carbon becomes negatively charged due to the deprotonation of functional groups and the adsorption of cationic pollutants will be favored.

In this study, the adsorption experiments were carried out at the natural pH of the 10 mg L^–1^ paracetamol solution (pH 5.6). Under these conditions, paracetamol (pKa = 9.38) is predominantly present in its neutral/protonated form,^51^ while the activated carbons exhibit slightly positive surface charge characteristics according to their pH_PZC_ values. Therefore, electrostatic interactions are not expected to be the predominant adsorption mechanism under the studied conditions. Similar results were reported by Nguyen and collaborators (2020) when studying the adsorption of paracetamol on commercial activated carbon.^52^ The authors pointed out that the removal process is little affected by variations in solution pH and ionic strength, suggesting that electrostatic interactions are not the dominant adsorption mechanism. Bouzidi et al. (2023) investigated the mechanisms of paracetamol adsorption on a new activated carbon synthesized from tree pods derived from Erythrina speciosa.^53^ The authors suggested that paracetamol adsorption occurs mainly through hydrogen bonding and π–π interactions between the drug molecules and the activated carbon surface. In addition, adsorbate multilayer formation may occur through hydrogen bonding or π–π stacking interactions.

Paracetamol adsorption can also be correlated with the porosity of activated carbons. The results of the present study suggest a relationship between the adsorption capacity of paracetamol by the materials and their surface area, since an increase in the removal of the organic molecule by AC is observed with the increase in the surface area promoted by the activation process. Bernal and collaborators (2017) studied the adsorption of paracetamol by modified activated carbons prepared from coconut shell.^51^ The authors concluded that the adsorption mechanism is governed by dispersive forces attributed to π–π electron interactions, since the oxygen functional groups capable of forming Lewis acid–base complexes or hydrogen bonds are absent in the studied activated carbons.

A tendency toward lower paracetamol adsorption was observed for activated carbons presenting lower pH_PZC_ values. This behavior may be associated with the higher surface acidity and polarity of these materials, resulting from the greater concentration of acidic functional groups on their surface. As the polarity increases, competition between water molecules and paracetamol molecules for the adsorption sites may occur.^54^ Since water molecules are more polar and more abundant in the reaction medium, the adsorption of paracetamol may be reduced. This behavior could partially explain the lower adsorption capacity observed for AC_waste, which presented the lowest pH_PZC_ value and surface area among the studied materials. Nevertheless, this interpretation should be considered cautiously, since adsorption experiments under different pH conditions were not performed in the present study.

In addition to the carbonaceous matrix, the residual inorganic phases identified by EDS, ICP-OES and TGA may also participate in the adsorption mechanism.^55^ Iron oxides have been reported to adsorb paracetamol through hydrogen bonding, electrostatic interactions and surface complexation involving hydroxylated Fe sites.^56^ Likewise, silica can adsorb paracetamol through silanol groups, while alumina surfaces may provide additional adsorption sites depending on their surface chemistry and solution pH,^55,57,58^ Nevertheless, the adsorption capacities reported for these individual inorganic materials are generally lower than those obtained for highly porous activated carbons.^55,57^ Therefore, considering the strong correlation observed in the present study between BET surface area and adsorption capacity, the porous carbon matrix is likely the dominant adsorbing phase, whereas the residual Fe-, Si- and Al-containing phases probably provide complementary adsorption sites rather than controlling the overall adsorption process.

Kinetic models are of great importance in adsorption studies. In addition to estimating the adsorption rate, they can also provide insights about the mechanisms involved in the process and how mass transport occurs in solution. Thus, the pseudo-first order,^59,60^ pseudo-second order^60^ and Weber-Morris (W&M—Intraparticle Diffusion)^61,62^ kinetic models were applied to the adsorption data. The parameters obtained for each model are shown in Table 2, and the graphs can be found in the Supplementary Material (Figure S3 and Figure S4). The best fit of the experimental data was expressed by comparing the correlation coefficient (R^2^), sum of squared prediction errors (SSE) and chi-square (X^2^).

**Table 2:** Adsorption Kinetic Parameters Calculated by Nonlinear Regression Kinetic Models

Kinetic model		Parameters	AC_Fe_2_O_3_	AC_soil	AC_waste
q_t_		*Experimental*	82.5	67.1	51.4
**Pseudo-first order** $q_{t}=q_{e}(1-e^{-k_{1}t})$		*k*_1_ (min^–1^)	0.314	0.182	0.047
		*q*_*e*_ (mg g^–1^) ± SD	75.3 ± 1.21	57.8 ± 1.09	42.8 ± 4.36
		*R*_1_^2^	0.990	0.991	0.949
		χ^2^	0.008	0.005	0.011
		*SSE*	0.053	0.032	0.078
**Pseudo-second order** $q_{t}=\frac{K_{2}{q_{e}}^{2}t}{1\;+\;k_{2}q_{e}t}$		*k*_2_ (g mg^–1^ min^–1^)	5.72	3.524	0.774
		*q*_*e*_ (mg g^–1^) ± SD	82.0 ± 1.42	65.8 ± 1.47	56.2 ± 6.86
		*R*_2_^2^	0.993	0.993	0.963
		χ^2^	0.005	0.003	0.008
		*SSE*	0.035	0.023	0.058
**W&M (Intraparticle Diffusion)** $q_{t}=k_{id}t^{1/2}$ + C	Step I	*k*_*id*_ (mg g^–1^ min^–1/2^)	17.2	13.2	4.66
		*Ci* (mg g^–1^)	17.1	3.56	3.93
		*R* ^2^	0.729	0.876	0.944
	Step II	*k*_*id*_ (mg g^–1^ min^–1/2^)	1.23	1.36	1.33
		*Ci* (mg g^–1^)	68.2	49.4	34.0
		*R* ^2^	0.889	0.956	0.946

For the application of the pseudo-first-order and pseudo-second-order models, the equilibrium data were not included in the treatment. As suggested by Simonin (2016), a fractional capture of the kinetic data of less than 85% was used.^63^ Both models showed a high correlation coefficient (R^2^) for the studied AC (Table 2). With the exception of AC_waste, the R_1_^2^ and R_2_^2^ values were very similar, with a higher R_2_^2^ value in all cases. Thus, other statistical parameters such as SSE and X^2^ were used to evaluate the performance of the kinetic models. The vales of SSE and X^2^ for the pseudo-second-order model were lower for AC_Fe_2_O_3_ and AC_soil. Thus, this model better described the data. Furthermore, the pseudo-second-order model also showed more satisfactory results for AC_waste, with a higher correlation coefficient when compared with the pseudo-first-order model (R_1_^2^ = 0.949 and R_2_^2^ = 0.963).

The intraparticle diffusion coefficient for the adsorption of paracetamol on activated carbons was obtained by plotting q­(mg g^–1^) *vs* t1/2 (Figure S4). The results suggest that the transfer of the adsorbate from the aqueous phase to the solid phase is a multistep process consisting of two types of mechanisms that reaffirm a two-step adsorption process. The values of C_i_ (mg g^–1^) estimate the thickness of the boundary layer. The higher its value, the greater the adsorption in the boundary layer. It can be observed that for all studied materials Step II shows higher adsorption values when compared with Step I. The second stage reflects a gradual adsorption, in which intraparticle diffusion is the limiting step.^64^ The proposed two-step mechanism suggests that the adsorbate is first deposited in the pores of the material surface due to the high surface area of the activated carbons. Subsequently, adsorbate-adsorbent interactions occur through hydrogen bonds or π–π stacking, as previously mentioned.

Figure 10 shows the adsorption isotherms of paracetamol by the studied activated carbons. As already observed in Figure 7, the adsorption isotherm confirms that AC_Fe_2_O_3_ showed a greater capacity to remove paracetamol, adsorbing 180 mg g^–1^ (Figure 10). It is also noted that the samples activated with soil and waste samples, AC_soil and AC_waste, adsorbed 114 and 94.6 mg g^–1^ of paracetamol, respectively. On the other hand, the adsorption capacity of the non-activated carbon (PCC) is only 5 mg g^–1^. These results show that the adsorption capacity of paracetamol by AC_Fe_2_O_3_, AC_soil and AC_waste are approximately 36, 23, and 19 times greater than that of PCC, which confirms the efficiency of the innovative activation process using iron oxides.

**Figure 10:**
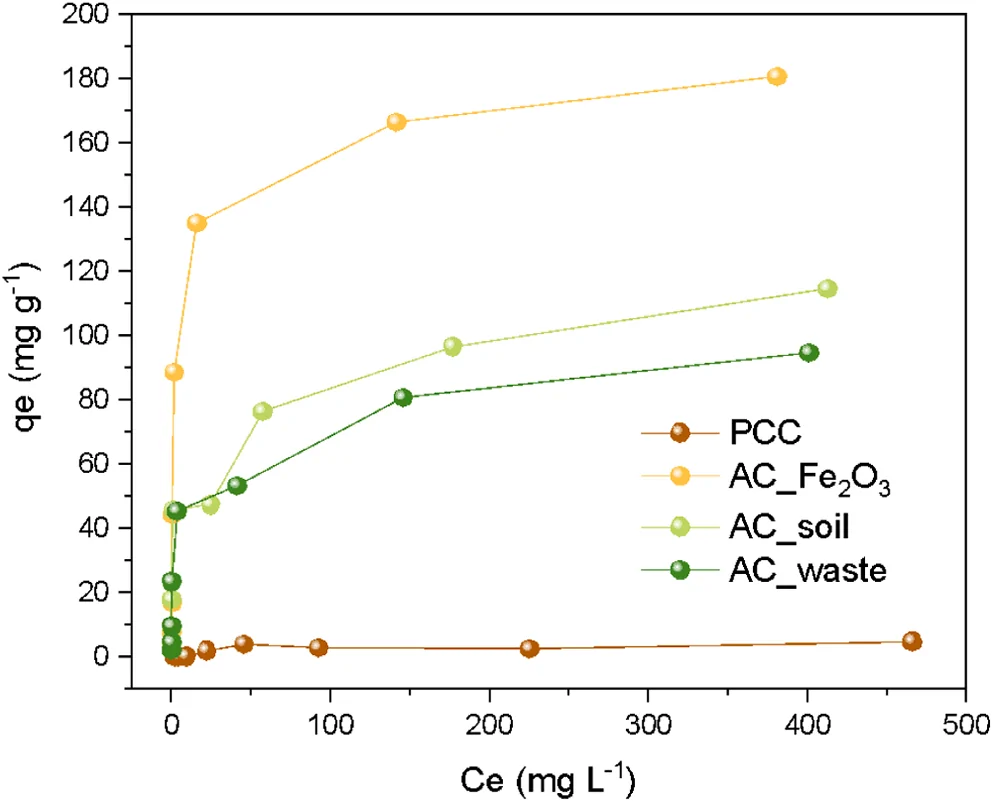
Adsorption isotherms of paracetamol onto AC and PCC.

Adsorption isotherms provide important physicochemical parameters on the relationship between adsorbate and adsorbent, critically improving the study of the behavior between the species involved in adsorption.^65^ The shape of the isotherms of the prepared ACs showed that adsorption increases with the increase in the concentration of paracetamol until reaching equilibrium. This shape suggests a behavior that allows the isotherms to be classified as type L2, which indicates that the adsorbent and adsorbate have a relatively strong affinity and that the chemisorption process prevails.^66^

The Langmuir and Freundlich isotherm models were used to study the adsorption of paracetamol by the activated carbons. The coefficients of determination (R^2^) and isothermal constants for the models are shown in Table 3. The graphs with the adjustments can be found in Figure S5 of the Supplementary File. For AC_Fe_2_O_3_, the equilibrium isotherm best fitted the Langmuir model, showing a maximum adsorption capacity very close to the experimental value. Thus, it agrees with the kinetic study adjusted for the pseudo-second-order model. Samples AC_soil and AC_waste showed a better fit to the Freundlich model. This model considers the adsorbent surface as heterogeneous and can be applied to multilayer adsorption.^67,68^ These results corroborate the differences observed in the N_2_ adsorption/desorption isotherms of the materials, which indicate multilayer adsorption on AC_soil and AC_waste.

**Table 3:** Parameters Calculated for the Freundlich and Langmuir Isotherms for the Adsorption Process of Paracetamol onto Activated Carbons

		Material		
Isotherm model	Parameters	AC_Fe_2_O_3_	AC_soil	AC_waste
Experimental value	*q*_*m*_ (mg g^–1^)	180	114	94.6
Freundlich $qe=K_{F}{C_{e}}^{1/nF}$	*K*_*F*_ (L g^–1^)	51.8	30.3	25.9
	*n*_*F*_	4.42 ± 0.92	4.54 ± 0.616	4.51 ± 0.578
	*1/n*_*F*_	0.226	0.220	0.222
	*χ* ^2^	811	80.3	57.2
	*R* ^2^	0.845	0.952	0.954
Langmuir $qe=\frac{q_{m}K_{L}C_{e}}{1\;+\;K_{L}C_{e}}$ $R_{L}=\frac{1}{1\;+\;K_{L}C_{0}}$	*q*_*m*_ (mg g^–1^)	170 ± 7.34	86.9 ± 11.1	78.6 ± 8.82
	*K*_*L*_ (L mg^–1^)	0.478	0.710	0.434
	*χ* ^2^	127	423	193
	*R* ^2^	0.975	0.749	0.845
	*R*_*L*_	0.455	0.360	0.479

The values obtained for the equilibrium parameters (1 > RL > 0) indicate a favorable adsorption, and the values of the diversity factor (1/nF) suggest a heterogeneous surface for the studied materials.

Finally, several studies have investigated the efficiency of different adsorbents for paracetamol removal from aqueous media. As shown in Table 4, the adsorption capacities reported in the literature vary considerably depending on the experimental conditions, such as pH, temperature, adsorbent dosage, and material preparation route. Therefore, the comparison presented herein should be considered only as a qualitative benchmark rather than a direct performance ranking. Within this context, the materials AC_soil and AC_waste demonstrated competitive adsorption performance compared with other activated carbons reported in the literature. Moreover, it is important to highlight that, unlike most studies that employed commercial high-purity precursors, the activated carbons developed in this work were produced from by-products, reinforcing their potential as low-cost and sustainable adsorbents. An important aspect that should be highlighted is the need to investigate the stability and reusability of these materials, as these parameters are essential for evaluating their practical applicability. Although such studies were not conducted in the present work, the results obtained suggest that AC_soil, AC_waste, and AC_Fe_2_O_3_ are promising adsorbents for the removal of paracetamol.

**Table 4:** Adsorption Capacities of Paracetamol onto Other Activated Carbons from the Literature

Adsorbent	S_BET_ (m^2^ g^–1^)	pH	Temperature (°C)	q_max_ (mg g^–1^)	k_f_ (L g^–1^)	Reference
AC_Fe_2_O_3_ (coffee cake)	614	5.6	25	170	51.8	Present study
AC_soil (coffee cake)	578	5.6	25	86.9	30.3	Present study
AC_waste (coffee cake)	458	5.6	25	78.6	25.9	Present study
ABSAC (sludge)	642	8.0	25	156.3	23.24	^68^
CAC (commercial activated carbon)	1248	7.0	25	221	90.1	^52^
WSAC (wood sawdust)	96.327	6.7	30	15.90	12.12	^70^
AC (commercial activated carbon)	866	8	25	93.97	11.62	^71^
SAC (biomass waste)	2794	6	30	356.22	120.68	^72^

### 3.4 Carbon Activation Reaction Mechanism

As described in the introduction, the process of carbon activation by iron oxide begins with carbothermic reduction (eqs 1–3). In this step, the reaction between the iron present in the oxides (α-Fe_2_O_3_, α-FeOOH, Fe_3_O_4_ and FeO) and the carbon structure promotes the reduction of Fe­(III) and Fe­(II) ions and, consequently, the oxidation of the carbon present on the coal surface. During this process, pores are formed, which initiates the coal activation process. The reactions involve the formation of carbon monoxide (CO) (eqs 1–3), which, at high temperatures, also reduces the iron oxides to metallic iron, resulting in the formation of carbon dioxide (CO_2_) (eqs 4–6). The CO_2_ formed, in turn, oxidizes the carbon (eq 7), contributing to the activation process by physical activation. During these reactions, carbon is consumed from the surface of the coal, which leads to the formation of pores, which are responsible for increasing the surface area of the material. It is important to highlight that, in this process, there is formation and consumption of CO/CO_2_, which actively participate in the redox reactions. Considering that the carbon surface is oxidized by these reactions and that CO and CO_2_ are produced and consumed throughout the process, the results suggest that this innovative methodology may involve a semi-closed carbon cycle (Figure 11).

**Figure 11:**
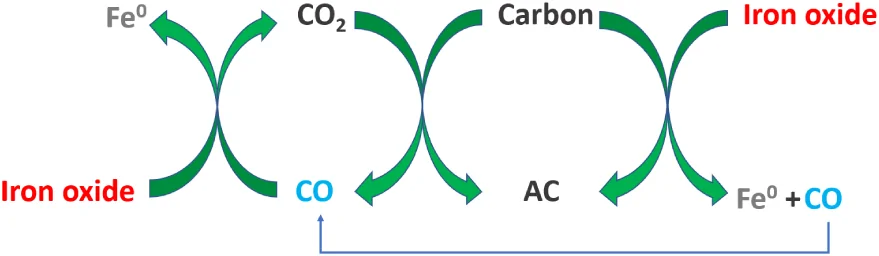
Carbon semicycle in the innovative coal activation process.

This hypothesis is supported by the results obtained in the work of Abanades and d’Izard (2025), in which the authors investigated the kinetics of hematite thermal reduction using different types of carbon materials mixed with commercial Fe_2_O_3_.^16^ The study demonstrated that carbon monoxide formation initially occurs according to the reaction presented in eq 1 and is subsequently consumed according to eq 4 Hematite reduction occurs through sequential stages (Fe_2_O_3_ → Fe_3_O_4_ → FeO → Fe) as the temperature increases inside the furnace. The formation of CO (eqs 1, 2, and 3) contributes to the Fe formation steps (eqs 4, 5, and 6), which are schematically represented at the ends of Figure 11. According to the results reported by Abanades and d’Izard (2025), the Boudouard reaction (C + CO_2_ → 2CO), which takes place at elevated temperatures, plays a fundamental role in CO production, promoting the final reduction step from FeO to Fe^0^ (eq 6).^16^

The proposed process shows the following interesting characteristics: (i) the production of two products in a single process, which may represent energy and cost savings; (ii) the process contributes to the circular economy, since industrial by-products can be used as sources of carbon and iron, such as biomass from the agroindustry and iron-rich mining waste; (iii) the possibility of using precursors that have already been carbonized in energy generation processes, such as soot generated in thermoelectric plants; and (iv) the CO and CO_2_ produced in the process may be involved in a semicyclic system, as illustrated in Figure 11.

## 4 Conclusion

The innovative activation process of pyrolyzed coffee bean cake (PCC) using low-cost agents such as mining waste, soil, and hematite proposed in this study has shown to be effective. XRD analysis indicated that iron, predominantly present in its trivalent form in the samples, was reduced to metallic iron. As a result, two products of interest were obtained: activated carbon and Fe^0^. In addition, the prepared activated carbons showed high paracetamol adsorption capacities, which can be attributed to the increase in the BET surface area of the activated materials. Among the studied materials, the activated carbon prepared from the commercial reagent hematite showed the highest paracetamol adsorption capacity and the largest surface area. This superior performance was related to the higher amount of Fe^3+^ present in the commercial reagent when compared with the Fe-rich mining waste and soil sample. Finally, this study resulted in the production of two materials with great potential for environmental applications, produced from accessible and abundant (by-) products, contributing significantly to the promotion of the circular economy and sustainability. Although the results demonstrate the technical feasibility of producing activated carbons using iron-rich activating agents, the industrial applicability of the process still requires validation through pilot-scale studies, including techno-economic assessment, process optimization, and large-scale operational evaluation.

## Supplementary Material



## Data Availability

The authors declare that the data supporting the findings of this study are available within the paper and its Supplementary Information files. Should any raw data files be needed in another format, they are available from the corresponding author upon reasonable request. Source data are provided with this paper.

## Notes

Additional experimental details: capsules used in the synthesis of carbonized coffee cake (Figure S1); X-ray diffractograms of the samples with reference patterns (Figure S2); kinetic models applied to the paracetamol removal curves (Figure S3); plots of the intraparticle diffusion model stages (Figure S4); isotherm models fitted to the paracetamol removal curves (Figure S5) (PDF)

## #

Federal Institute of Goiás, IF-Goiano, ZIP Code 73900 -000, Posse, State of Goiás, Brazil

## ∇

Department of Engineering, Federal University of Lavras, ZIP code 37203 -202, Lavras, State of Minas Gerais, Brazil.

## ○

Department of Soil Science, Federal University of Lavras, ZIP code 37203 -202, Lavras, State of Minas Gerais, Brazil.

## †

Organic Chemistry Laboratory – Essential Oils, Department of Chemistry, Federal University of Lavras, 37203-202 Lavras, State of Minas Gerais, Brazil
