# Supplementary material for: Activated Carbon Production from Coffee Waste Employing Soil, Mining Waste, and Hematite as Activating Agents
Source: ACS Omega. 2026 Oct 6;11(41):62443–57. doi: 10.1021/acsomega.6c00989 (PMC13689301; doi:10.1021/acsomega.6c00989)
Supplement: Supplementary file 1 [file ao6c00989_si_001.pdf]

SUPPLEMENTARY FILE

ACTIVATED CARBON PRODUCTION FROM COFFEE WASTE EMPLOYING SOIL, MINING WASTE AND HEMATITE AS ACTIVATING AGENTS

Pricila M. B. Chagas[a1], Aline A. Caetano[a2], Evanise S. Penido[a3], Mozarte S. Santana[a], Maria das G. Cardoso[a], Fabiano Magalhães[a]*
Department of Chemistry, Federal University of Lavras, Zipcode 37203-202, Lavras, State of Minas Gerais, Brazil.
*E-mail: fabianomagalhaes@ufla.br (Fabiano Magalhães)

*Corresponding author

Fabiano Magalhães

Department of Chemistry, Federal University of Lavras

CEP 37203-202, Lavras, MG, Brazil.

Phone: +(55) 35 99914-0193

e-mail: fabianomagalhaes@ufla.br

[1] Federal Institute of Goiás, IF-Goiano, Zipcode 73900-000, Posse, State of Goiás, Brazil.
[2] Department of Engineering, Federal University of Lavras, Zipcode 37203-202, Lavras, State of Minas Gerais, Brazil.
[3] Department of Soil Science, Federal University of Lavras, Zipcode 37203-202, Lavras, State of Minas Gerais, Brazil.

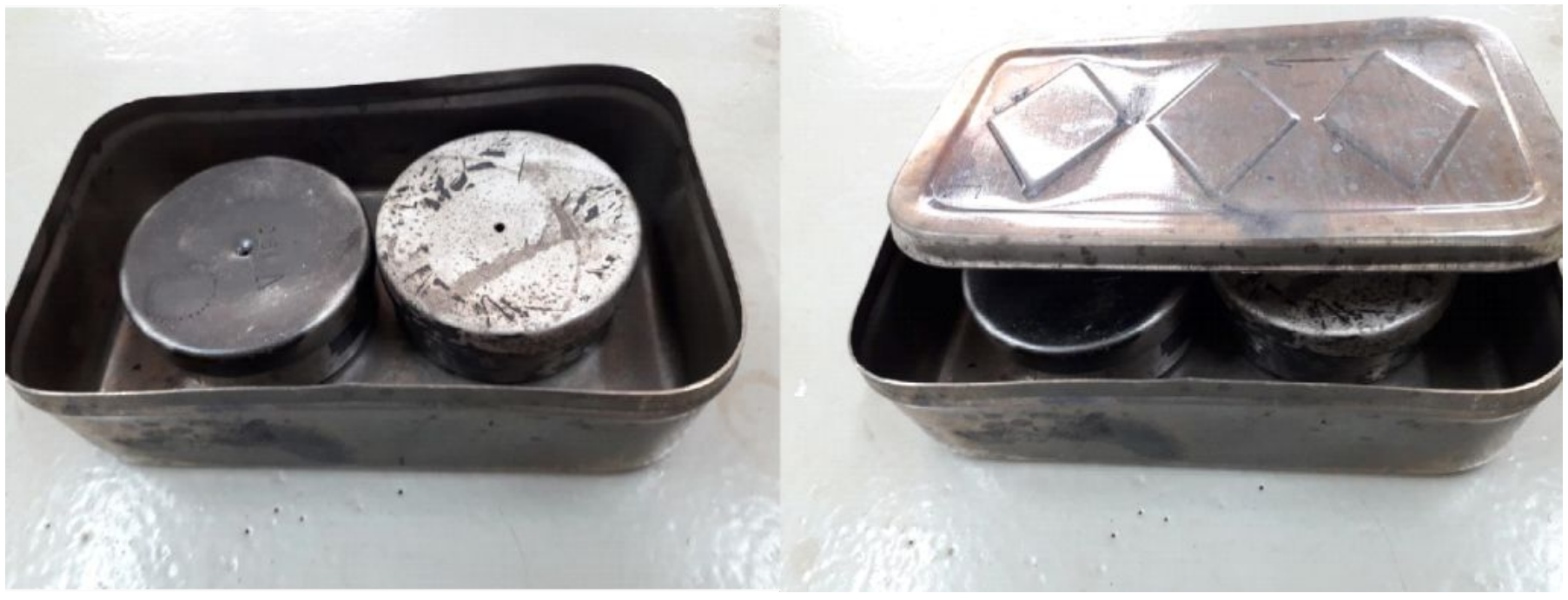

Figure S1 Capsules used in the synthesis of carbonized coffee cake (CC).

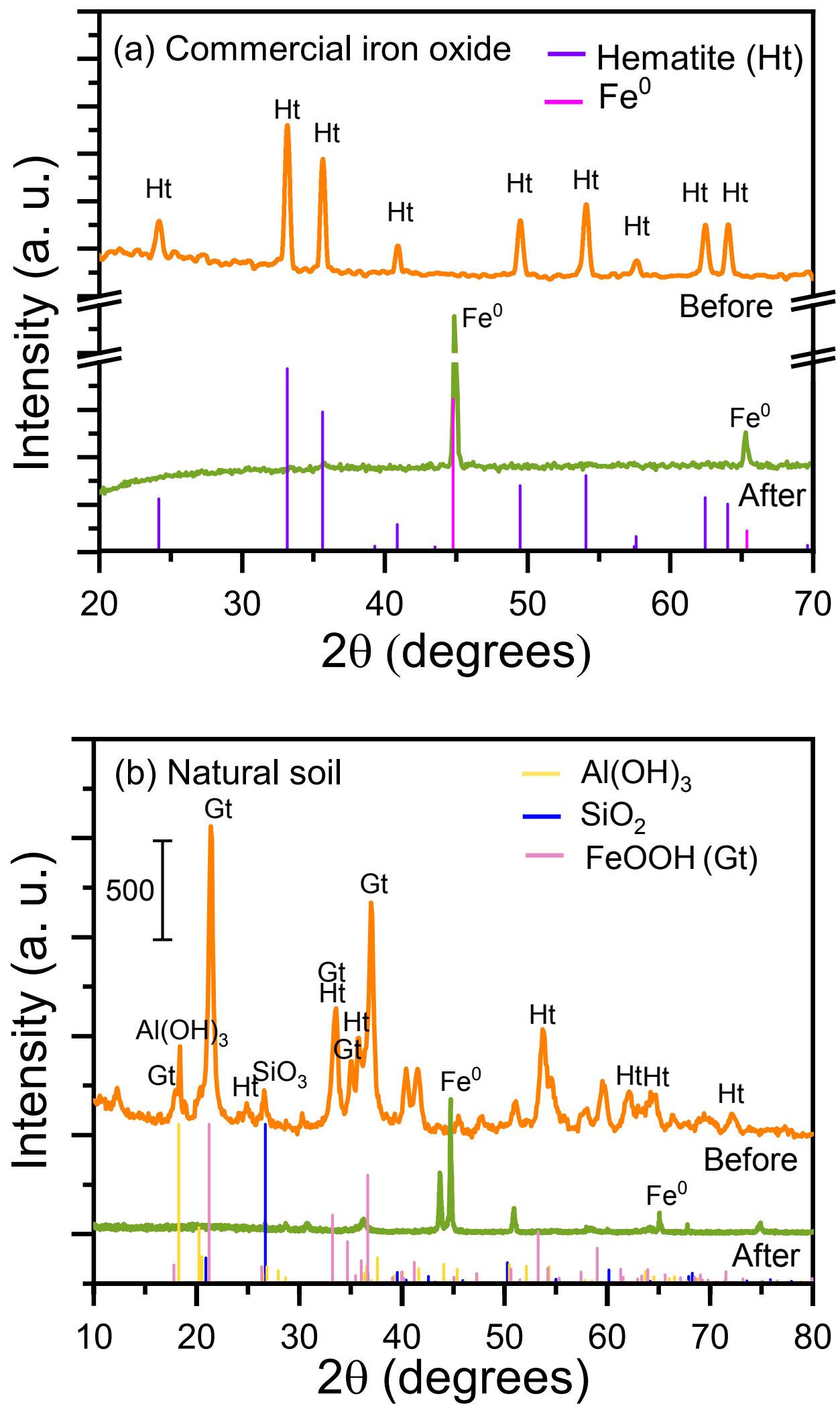

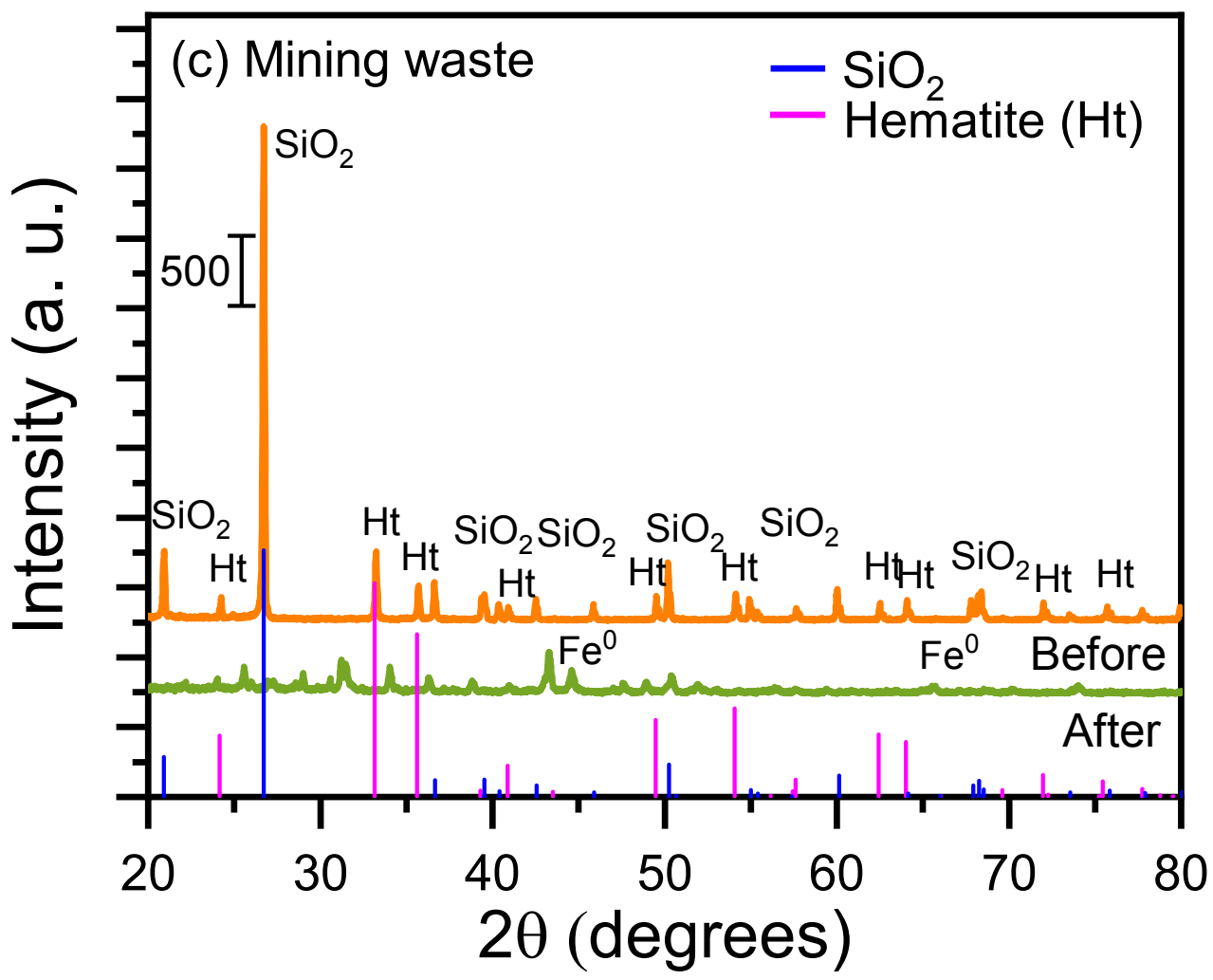

Figure S2 X-ray diffractograms for the samples (a) commercial iron oxide (b) soil and (c) mining residue before and after the activation process, along with reference patterns.

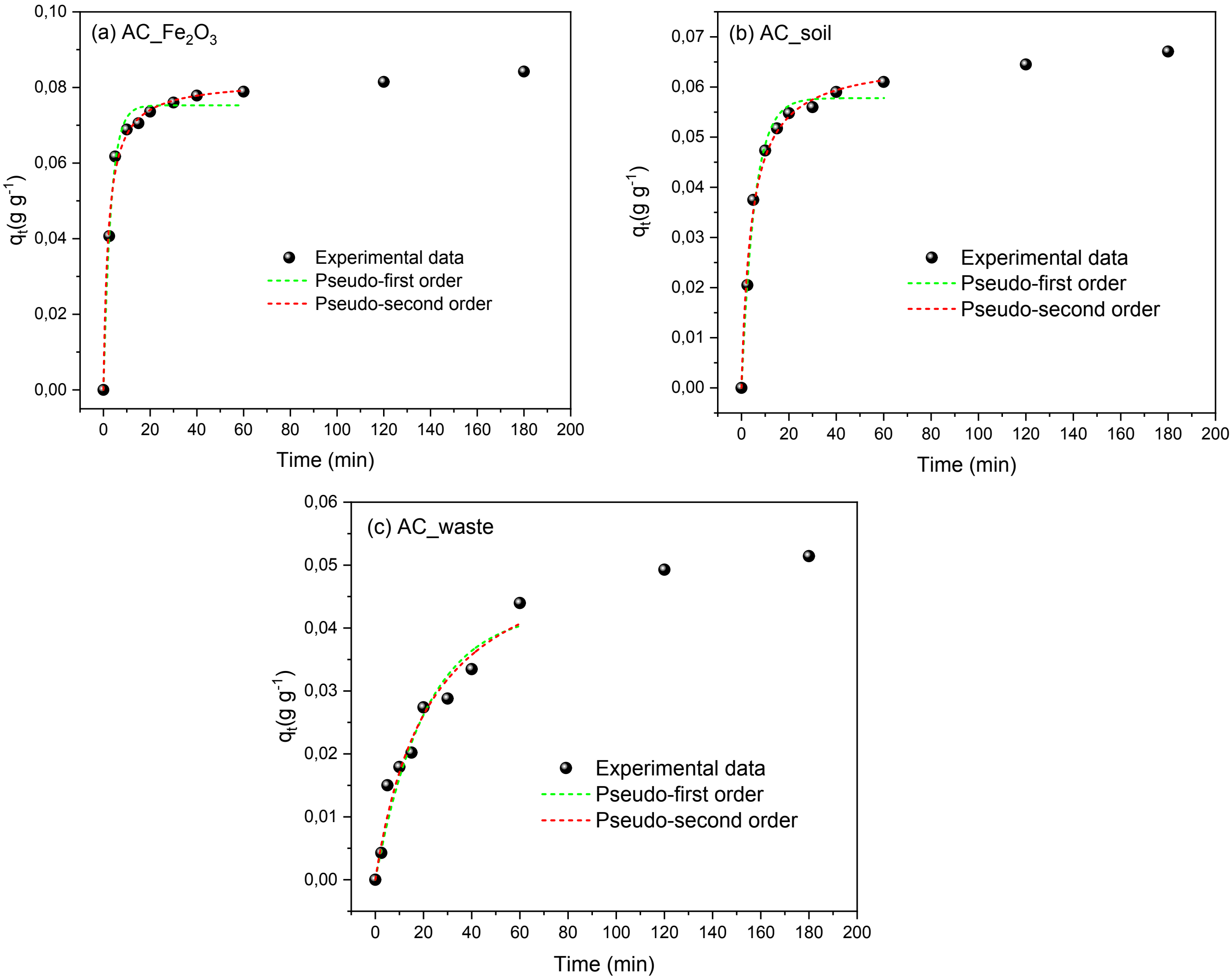

Figure S3 Kinetic models applied to the removal curves of paracetamol.

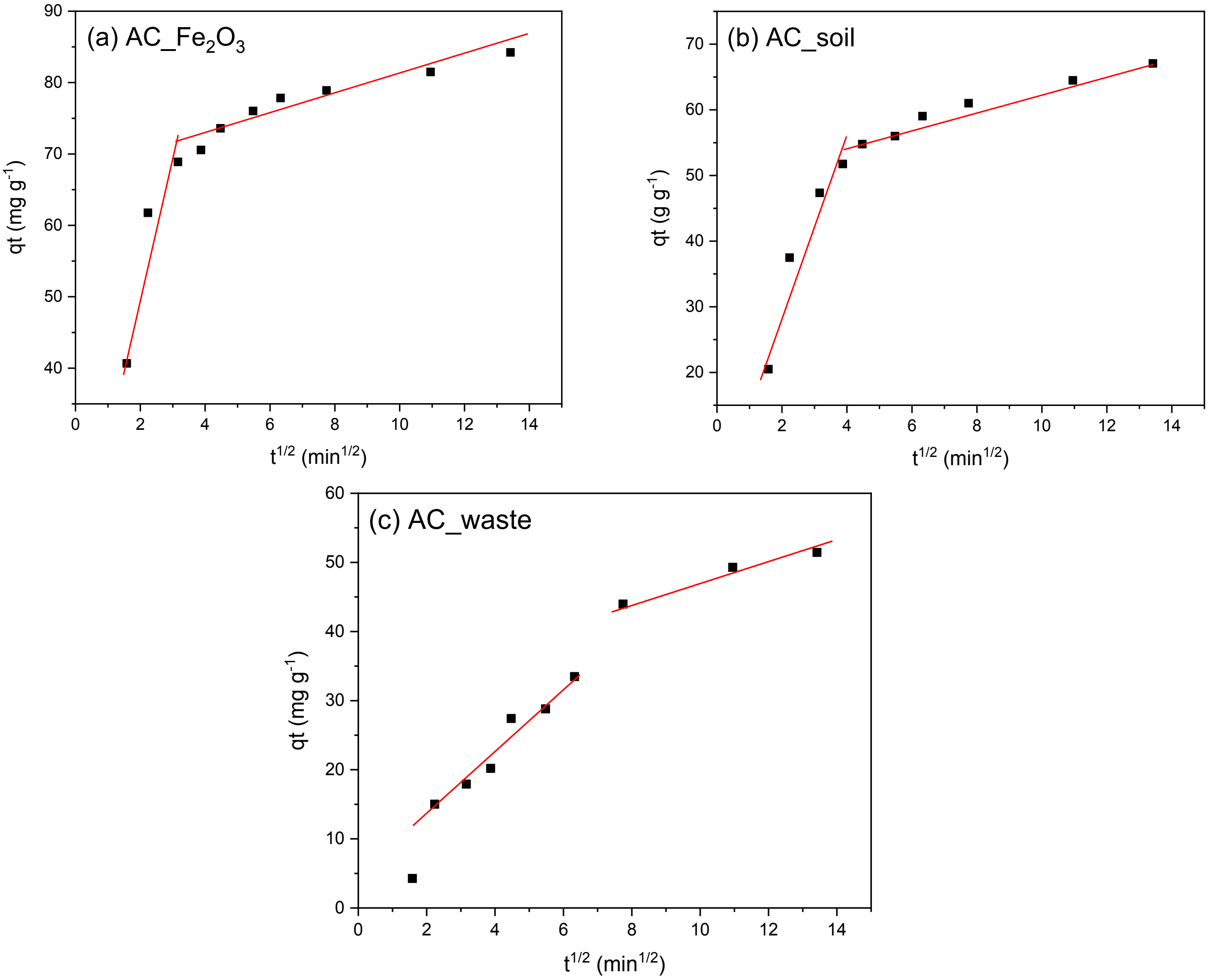

Figure S4 Plots of the Intraparticle Diffusion model stages.

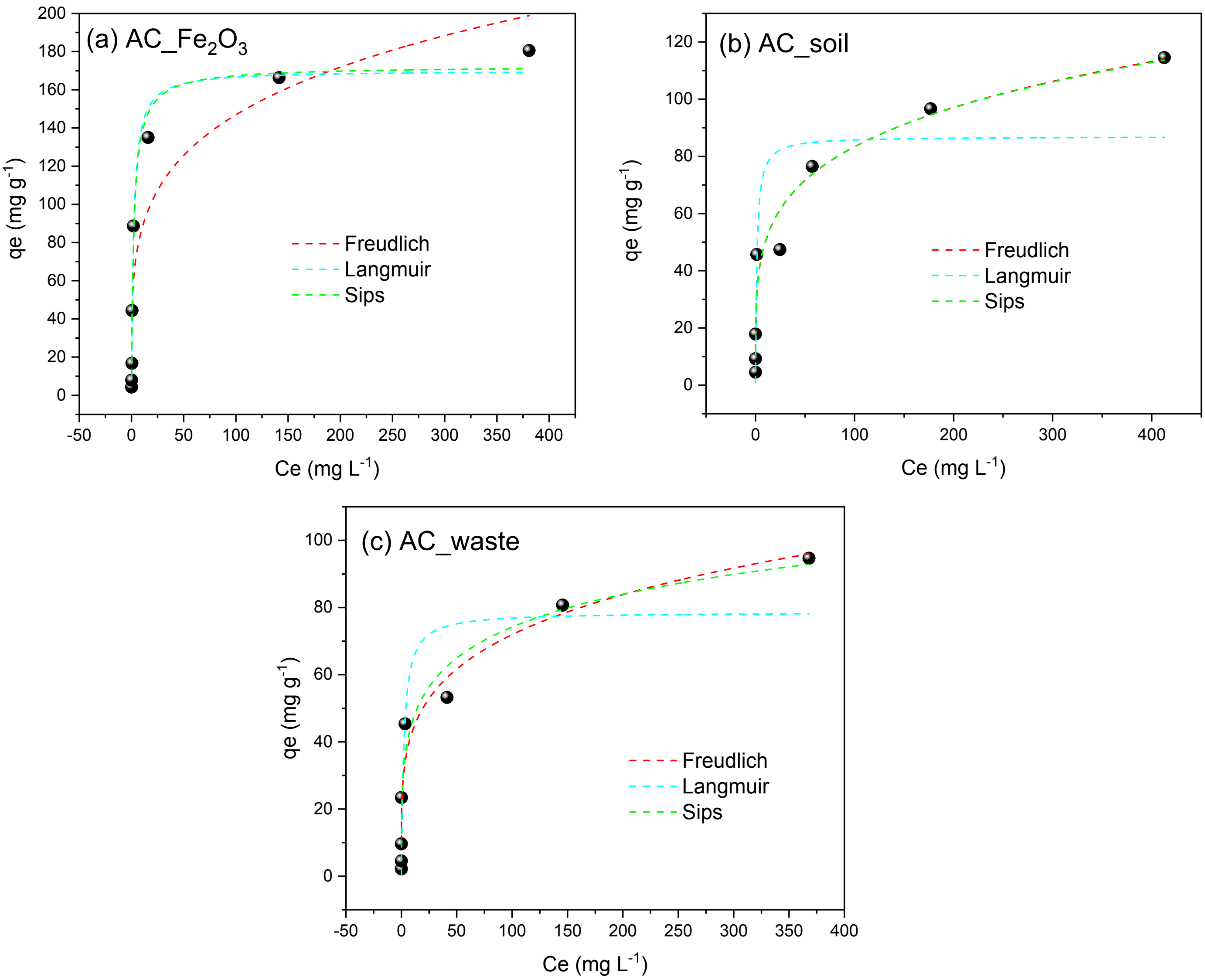

Figure S5 Isotherm models fitted to the paracetamol removal curves.
